# Enhanced Expression of *miR-181b* in B Cells of CLL Improves the Anti-Tumor Cytotoxic T Cell Response

**DOI:** 10.3390/cancers13020257

**Published:** 2021-01-12

**Authors:** Mirco Di Marco, Serena Veschi, Paola Lanuti, Alice Ramassone, Stefania Pacillo, Sara Pagotto, Felice Pepe, Jonahunnatha Nesson George-William, Claudia Curcio, Marco Marchisio, Sebastiano Miscia, Idanna Innocenti, Francesco Autore, Barbara Vannata, Patrizia Di Gregorio, Mario Di Gioacchino, Silvia Valentinuzzi, Manuela Iezzi, Renato Mariani-Costantini, Luigi Maria Larocca, Luca Laurenti, Angelo Veronese, Rosa Visone

**Affiliations:** 1 Center for Advanced Studies and Technology (CAST), G. d’Annunzio University, 66100 Chieti, Italy; dmamirco@gmail.com (M.D.M.); veschi@unich.it (S.V.); p.lanuti@unich.it (P.L.); alice.ramassone@unich.it (A.R.); stefania.pacillo28@gmail.com (S.P.); sara.pagotto@unich.it (S.P.); felixpepe@hotmail.it (F.P.); jonahnesson@gmail.com (J.N.G.-W.); claudia.curcio@unito.it (C.C.); m.marchisio@unich.it (M.M.); m.digioacchino@unich.it (M.D.G.); valentinuzzis@gmail.com (S.V.); miezzi@unich.it (M.I.); rmc@unich.it (R.M.-C.); a.veronese@unich.it (A.V.); 2 Department of Medical, Oral and Biotechnological Sciences, G. d’Annunzio University, 66100 Chieti, Italy; 3 Department of Medicine and Aging Sciences, G. d’Annunzio University, 66100 Chieti, Italy; sebastiano.miscia@unich.it; 4 Fondazione Policlinico Universitario A. Gemelli IRCCS, 00168 Rome, Italy; idanna.innocenti@yahoo.it (I.I.); francesco_autore@yahoo.it (F.A.); bvannata@hotmail.it (B.V.); Luca.Laurenti@unicatt.it (L.L.); 5 Institute of Transfusion Medicine, “Ss. Annunziata” Hospital, 66100 Chieti, Italy; patrizia.digregorio@asl2abruzzo.it; 6 Institute of Pathological Anatomy, Fondazione Policlinico Universitario A. Gemelli IRCCS t, 00168 Rome, Italy; luigimaria.larocca@unicatt.it (L.M.L.)

**Keywords:** CLL, microRNA, *miR-181b*, cytotoxic T cells, CD40-CD40L

## Abstract

**Simple Summary:**

Low expression of *miR-181b* in chronic lymphocytic leukemia (CLL) is linked to progression and cell death resistance. Patients with the progression of the disease show immune dysfunction. We aim at studying whether *miR-181b* is also involved in this process. We demonstrate that *miR-181b* can be increased in CLL cells by crosstalk with CD40L^+^ T cells, enhancing the maturation of CD8^+^ T cells in cytotoxic T lymphocytes (CTL) and, in turn, the anti-tumor cytotoxic T cell response in in vitro and in vivo models. These results demonstrate a role of the *miR-181b* in the immune response against tumor and envisage a therapeutic action of *miR-181b* in a specific milieu surrounding CLL cells.

**Abstract:**

The clinical progression of B cell chronic lymphocytic leukemia (CLL) is associated with immune cell dysfunction and a strong decrease of *miR-181b-5p* (*miR-181b*), promoting the death of CLL cells. Here we investigated whether the reduction of *miR-181b* impairs the immune response in CLL. We demonstrate that activated CD4+ T cells increase *miR-181b* expression in CLL through CD40–CD40L signaling, which enhances the maturation and activity of cytotoxic T cells and, consequently, the apoptotic response of CLL cells. The cytotoxic response is facilitated by a depletion of the anti-inflammatory cytokine interleukin 10, targeted by *miR-181b*. In vivo experiments in NOD.Cg-Prkdcscid Il2rgtm1Wjl/SzJ mice confirmed that *miR-181b* promotes the apoptotic death of CLL cells only when functional T cells are restored. Overall, our findings suggest that the reinstatement of *miR-181b* in CLL cells could be an exploitable adjuvant therapeutic option for the treatment of CLL.

## 1. Introduction

Cancer cells use several mechanisms to elude attacks by the immune system [1]. In Chronic Lymphocytic Leukemia (CLL), immune evasion can be caused by intrinsic defects that make B cells resistant to death and/or by poorly functioning T cell-mediated immune responses. In particular, T cells from CLL patients show impaired ability to interact with CLL cells [2,3,4] and acquired CD40L deficiency upon contact with CLL cells [5]. In a physiological scenario, CD40L^+^CD4^+^ T cells may induce opposite fates in B cells: (1) Clonal proliferation when foreign antigens stimulate the B cell receptor (BCR), and (2) clonal depletion when the BCR is either unbound or binds self-antigens or presents defective signaling [6,7]. T cell expressing CD40L induces mitogenic signals in B cells but also the expression of CD95 (FAS), a death signal [8].

BCR signaling is constitutively activated in CLL cells [9]; therefore, stimulated CD40L+ T cells should prime B cells to apoptosis instead of supporting clonal expansion [10]. An additional mechanism by which CLL cells evade surveillance is their capacity to secrete immunosuppressive cytokines, such as interleukin 10 (IL10), which interferes with T cell activation [11,12].

MicroRNAs (miRNAs) take part in the immunological key processes of innate and adaptive immune cells [13], and their deregulation contributes to the pathogenesis of CLL [14]. Specifically, *miR-181b*, a regulator of B cell differentiation, is expressed at low levels in CLL cells [15], which at least partly determines high levels of its targets, the antiapoptotic proteins MCL1 and BCL2, and consequently the resistance to proapoptotic drugs [16,17]. We previously reported that the levels of *miR-181b* decrease during disease progression in peripheral blood mononuclear cells (PBMCs) from CLL patients [17]. Moreover, in a CLL murine model, *miR-181b* reduces the leukemic cell fraction [18]. *MiR-130a* and *miR-181a* are also able to induce the death of primary CLL cells in vitro [19], and their expression similarly decreases during CLL progression [20].

We hypothesized that the low expression of *miR-181b, miR-181a,* and *miR-130a* that occur in patients during CLL progression could be due to the impaired interactions between T and CLL cells. To this end, we tested whether, potentiating such interactions, the expression of *miR-181b, miR-181a,* and *miR-130a* could be restored and whether restoring the expression of *miR-181b* could promote cell-mediated immune response and apoptosis in CLL cells.

## 2. Results

### 2.1. Activated T Cells Increase the Expression of miR-181b, miR-181a and miR-130a in CLL Cells

To investigate whether the decreased expression of *miR-181b*, *miR-181a*, and *miR-130a* observed in CLL could reflect inefficacy of T cell activation, we co-cultured CLL cells with CD4^+^T cells from healthy donors, since T cells from CLL patients show pathological defects [2,21]. Resting T cells were activated using antibodies against CD2, CD3, and CD28 and co-cultured with purified primary CLL cells. CD95 expression was analyzed to control B cell activation upon T/B cell interaction [22] (Figure S1).

**Figure 1:**
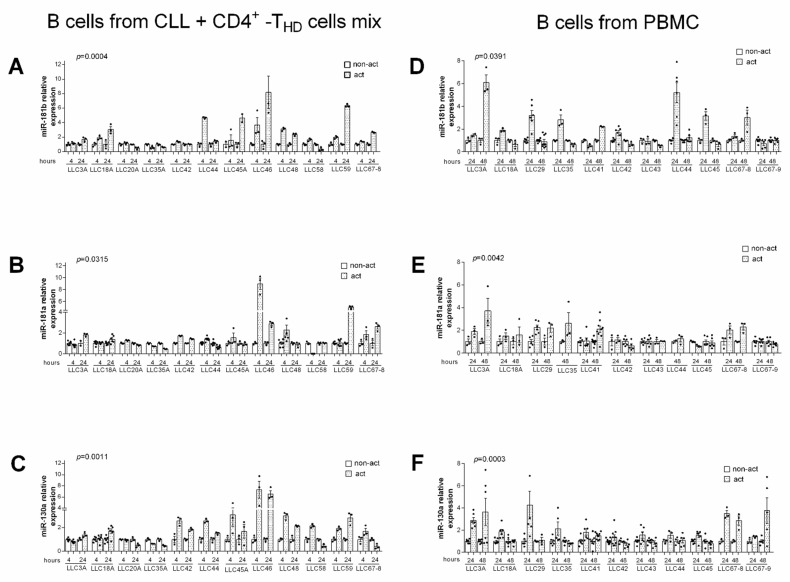
Exogenous activation of T cells increases *miR-181b*, *miR-181a,* and *miR-130a* expression levels in Chronic Lymphocytic Leukemia (CLL) cells. (**A**–**C**) Relative gene expression values in purified CLL cells co-cultured with activated vs. non-activated T cells. Purified CLL cells were mixed with either non-activated or activated (by anti-CD2, -CD3, -CD28 antibodies) T cells (CD4+) from healthy donors (HD) at a T/B ratio of 1:1. After 4 and 24 h CLL were re-purified and assayed for gene expression. (**D**–**F**) Relative gene expression values in purified CLL cells isolated from activated or non-activated peripheral blood mononuclear cells (PBMCs) from CLL patients. PBMC were activated as described above and then cultured for an additional 24 or 48 h. Relative expression values were determined by RT-qPCR; miRNAs data were normalized to the endogenous references *RNU44* with the 2^−Δct^ method. For each patient, the relative expression of miRNAs was normalized to the level of non-activated sample. Data are presented as means ± SEM and technical replicates are shown for each sample as black dots (●). *p*-values were calculated using Wilcoxon test in activated vs. non-activated CLL cells.

The analysis of re-purified CLL cells after 4 or 24 h of incubation with activated allogenic T cells revealed significantly increased expression of *miR-181b*, *miR-181a,* and *miR-130a* (*p* = 0.0004, *p* = 0.0315, and *p* = 0.0011 respectively, Wilcoxon test) (Figure 1A–C). In a similar experiment conducted using PBMCs from CLL patients, we found that *miR-181b*, *miR-181a*, and *miR-130a* significantly increased in purified CLL cells after 24 or 48 h of PBMC activation (*p* = 0.0391, *p* = 0.0042, and *p* = 0.0003 respectively, Wilcoxon test) (Figure 1D–F).

Given the low T cell fractions in PBMCs from CLL patients, purified B cells from three CLL patients were co-cultured in 1:1 or 1:2 ratios with either autologous or allogenic T cells from CLL patients. In all cases, co-culture with stimulated CD4^+^T cells increased *miR-181b* expression in CLL cells (Figure S2), indicating that T cells from CLL patients retain the ability to up-regulate *miR-181b* upon exogenous activation.

**Figure 2:**
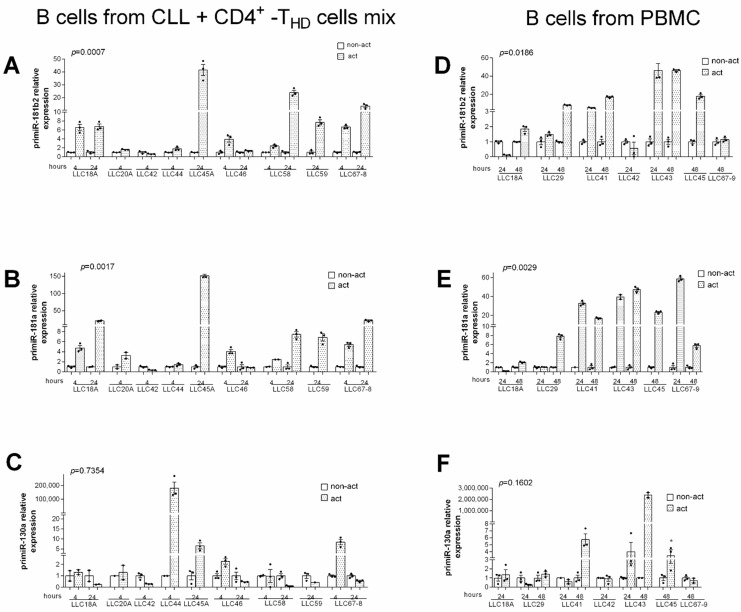
Exogenous activation of T cells increases *pri-miR-181b* and *pri-miR-181a* expression levels in CLL cells. (**A**–**C**) Relative gene expression values in purified CLL cells co-cultured with activated vs. non-activated T cells. Purified CLL cells were mixed with either non-activated or activated (by anti-CD2, -CD3, -CD28 antibodies) T cells (CD4+) from healthy donors (THD) at a T/B ratio of 1:1. After 4 and 24 h CLL were re-purified and assayed for gene expression. (**D**–**F**) Relative gene expression values in purified CLL cells isolated from activated or non-activated PBMCs from CLL patients. PBMCs were activated as described above and then cultured for additional 24 or 48 h. Relative expression values were determined by RT-qPCR; pri-miRNAs data were normalized to the endogenous references *ACTB* with the 2^−Δct^ method. For each patient, the relative expression of pri-miRNAs was normalized to the level of non-activated sample. Data are presented as mean ± SEM and technical replicates are shown for each sample as black dot (●). *p*-values were calculated using Wilcoxon test in activated vs. non-activated CLL cells (* *p* < 0.05).

To evaluate if *miR-181b*, *miR-181a,* and *miR-130a* increased at the transcriptional level, we analyzed the expression of their pri-miRNAs in several cases. We found that in CLL cells, the *pri-miR-181b2* and *pri-miR-181a* were controlled by exogenously activated CD4^+^T cells, both allogenic (*p* = 0.0007 and *p* = 0.0017 respectively, Wilcoxon test) (Figure 2A,B) and autologous (*p* = 0.0186 and *p* = 0.0029 respectively, Wilcoxon test) (Figure 2D,E). This was not the case for *pri-miR-130* (Figure 2C,F).

### 2.2. Transcriptional Up-Regulation of miR-181b and miR-130a after CD40–CD40L Interaction

**Figure 3:**
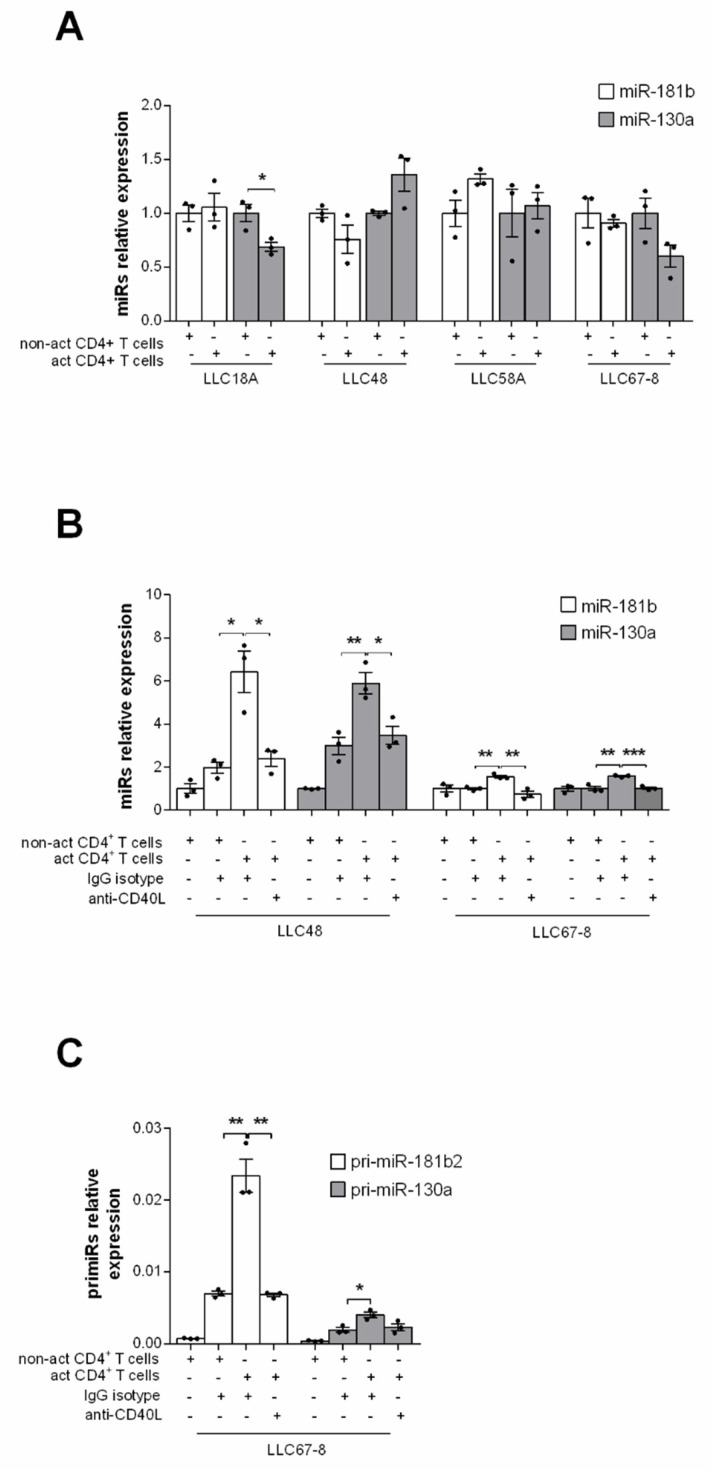
*MiR-181b* and *miR-130a* are up-regulated by CD40–CD40L interactions. (**A**) Relative expression by RT-qPCR of *miR-181b* and *miR-130a* in pure CLL cells co-cultured in a transwell with either activated or non-activated healthy CD4+ T cells for 4 h. B cells were seeded in the upper and T cells in the lower chamber (ratio T:B, 1:1). Expression data were obtained from four different patients. (**B**) *MiR-181b* and *miR-130a* and (**C**) *pri-miR-181b* and *pri-miR-130a* relative expression by RT-qPCR in purified CLL cells from CLL patients after 4 h of co-culture with either activated or non-activated healthy CD4+ T (ratio T:B, 1:1). T cells were pre-incubated with anti-CD40L antibody or the control IgG isotype. (**C**) Data are presented as mean ± SEM and technical replicates are shown for each sample as black dot (●). *p*-values were calculated using Student’s *t* test (* *p* < 0.05, ** *p* < 0.01 and *** *p* < 0.001).

We next investigated whether direct contact between T and CLL cells was necessary to increase the expression of the studied miRNAs. To this end, CD4^+^T cells from healthy donors, activated or not, were co-cultured in a transwell system with patient-derived CLL cells from the above experiment (Figure 1). The levels of *miR-181b* and *miR-130a* did not increase in CLL cells in all the tested cases suggesting that direct B-T cells contact is required to up-regulate this microRNA (Figure 3A). To assess whether CD40-CD40L signaling, defective in CLLs [23], could explain the effect B-T cells interactions on *miR-181b* and *miR-130a*, we co-cultured CLL cells with either exogenously activated or non-activated CD4^+^T cells pre-incubated with anti-CD40L monoclonal antibody (mAb) to block CD40-CD40L interactions (Figure S3) or with the isotype control. The T cell-mediated effect on both *miR-181b* and *miR-130a* was significantly inhibited at the transcriptional level by the anti-CD40L antibody (Figure 3B), as confirmed by pri-miRNAs analysis (Figure 3C).

Overall, these results demonstrate that in CLL cells, the *miR-181b* and *miR-130a* levels are affected by T cell signaling. Hereafter, we focused on *miR-181b* because of its stronger reported correlation with CLL progression [17].

### 2.3. MiR-181b Expression Pattern in Lymph Nodes of CLL Patients

**Figure 4:**
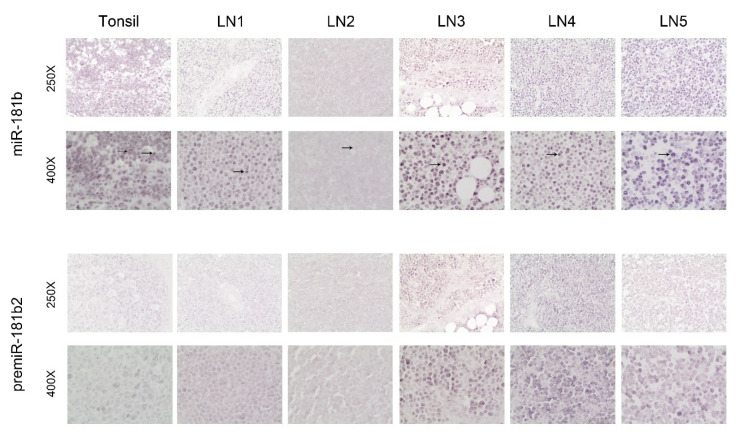
In situ hybridization of *miR-181b* and the respective pre-miRNA on tissue sections from tonsil of non-cancer patients and from lymph nodes of CLL patients. One representative picture of a tissue section of tonsil is shown (upper left) and lymph node sections from 5 CLL patients at 250X and 400X magnifications. Bold arrows indicate the nuclear localization, while slim arrows the cytoplasmic localization.

Since CD40 signaling is activated in lymph nodes from CLL patients and in tonsils of non-cancer patients [24,25,26], we performed ISH analysis in five lymph nodes from five CLL patients (LN1-LN5) and in two tonsils from two non-cancer patients. In the tonsils, we detected both nuclear and cytoplasmic *miR-181b* expression, with a gradual decrease in intensity from the dark to the light zone of the germinal centers (data not shown), as previously described [27]. In lymph nodes from CLL patients, we observed almost exclusively nuclear *miR-181b* staining (Figure 4, top rows); with somewhat lower intensity in two cases (LN1 and LN2). RT-qPCR on RNA from CLL cells purified from PBMCs confirmed *miR-181b* expression mainly in the nuclear fractions (Figure S4). Pre-miRNA expression was also analysed by ISH in lymph nodes. The localization of *pre-miR-181b2* was mostly nuclear, with a pattern similar to that of *miR-181b* (Figure 4, bottom row). The content of T cells in the close sections of these samples is shown (Figure S5). These data confirm that cytoplasmic *miR-181b* is almost undetectable in leukemic cells from CLL patients and indicate that reduced transcription of the miRNA gene and defective post-transcriptional processing affect *miR-181b* expression and localization in CLL cells.

### 2.4. Increased Expression of miR-181b in CLL Cells Enhances the Cytotoxic Activity of T Lymphocytes

The crucial role of CD40 signaling in triggering B cells deletion by cytotoxic T lymphocytes [6] prompted us to investigate the involvement of *miR-181b* in this process. GFP-fluorescent lentiviruses expressing either *miR-181b* (LV miR-181b) or control (LV CTRL) were generated and tested (Figure S6A). The CLL cell line MEC-1 was transduced with either LV miR-181b or LV CTRL and mixed with allogenic healthy CD8^+^T cells. We consistently observed a slight increase of late apoptosis in *miR-181b*-transduced MEC-1 cells as compared to the controls (Figure S7), suggesting that *miR-181b* increased the sensitivity of CLL cells to effector lymphocytes.

**Figure 5:**
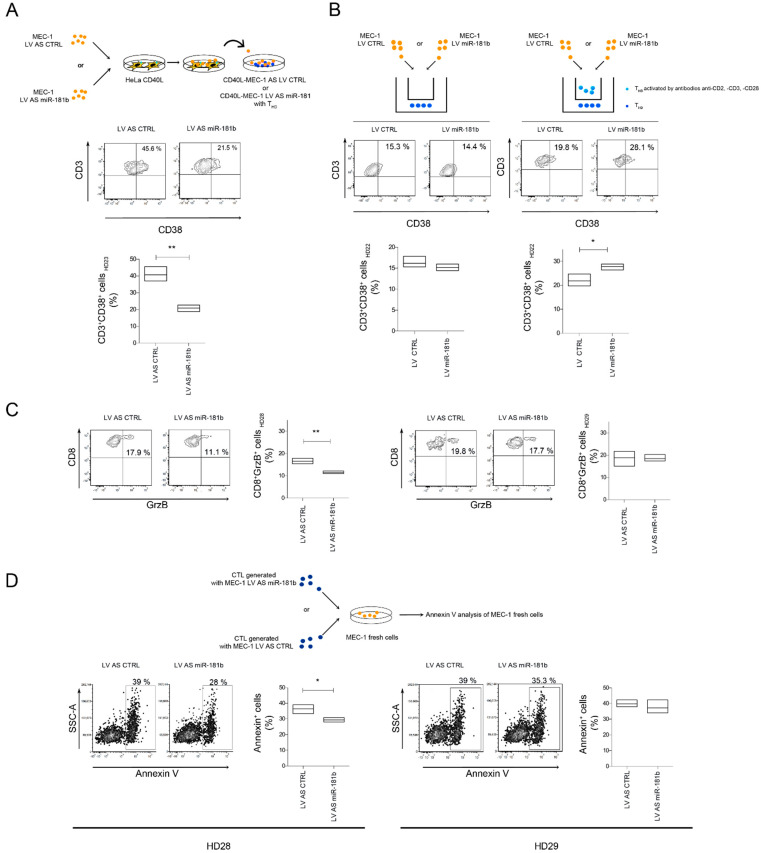
*MiR-181b* promotes the death of leukemia cells by inducing cytotoxic lymphocytes (CTL) maturation. (**A**) MEC-1 cells infected with either LV AS miR181b or LV AS CTRL were co-cultured with CD40L-HeLa. After three days, stimulated and transduced MEC-1 cells were mixed with CD3+ cells from healthy donor 23 (T_HD23_) to generate CTLs. Percentage of CD38+/CD3+ cells were measured after 8 days of co-culture. (**B**) MEC-1 cells infected with either LV miR-181b or LV CTRL were cultured with or without healthy activated T cells from healthy donor 22 (T_HD22_) (E/T ratio, 1:9) in the upper transwell chamber. In the lower chamber, T cells from the same healthy donors were seeded. After eight days, percentage of CD38+/CD3+ cells were measured in the lower transwell chamber. (**C**) Percentage of CD8+/GrZB+ cells from healthy donors 28 and 29 (T_HD28_ and T_HD29_) after generation of effector CTL assay as described in B. (**D**) Percentage of fresh VPD+/AnnV+ MEC-1 cells co-cultured for 1 h with CTLs generated in D; E/T ratio, 2:1. Data (**A**–**D**) are reported as floating bars (min to max) with the central line marking the mean of experimental triplicates. *p*-values were calculated by Student’s t test (* *p* < 0.05, ** *p* < 0.01).

To further investigate the role of *miR-181b* in the cell-mediated immune response, we generated cytotoxic T lymphocytes (CTLs) by mixing CD40L-stimulated MEC-1 and healthy allogenic T cells [28]. Before stimulation with Hela-CD40L, MEC-1 cells were transduced with a tested lentivirus expressing antisense *miR-181b* (LV AS miR-181b) (Figure S6B). After co-culture, T cells were assessed for CD38, linked to immune response activation and cytotoxic activity [29,30]. *MiR-181b* silencing in CLL cells led to a significant reduction of CD38^+^ T cells compared to the control (Figure 5A). To assess whether *miR-181b* needed direct T-B cells contact to exert its effect, MEC-1 cells infected with either LV CTRL or LV miR-181b were grown in upper transwell chambers, alone or with activated allogenic healthy T cells; while pure, non-activated T cells from the same healthy donor were seeded in the lower chambers. Activated T lymphocytes were used to boost T-B cell interactions. *MiR-181b* increased the CD38^+^ T cell fraction in the lower chamber in the presence of direct T-B cell contact in the upper chamber (Figure 5B). Accordingly, in a similar experiment, inhibition of *miR-181b* decreased the CD38^+^ T cell fraction and the percentages of T cells expressing granzyme B (GrzB) (Figure S8A). Next, we measured granzyme B expression in CD8^+^ T cells from two healthy donors (HD28, HD29) after the CTL generation assay. We noted that in one of the two-studied case, CTLs were less abundant when T cells were co-cultured with *miR-181b*-depleted MEC-1 cells, and T lymphocytes had lower cytotoxic activity when mixed with fresh third-party MEC-1 cells (Figure 5B,D). Variability of the results could be due to a different HLA matching between B and T allogenic cells. Similarly, CTLs from healthy donor 22 (HD22), generated with *miR-181b*-depleted MEC-1, induced weaker apoptosis in fresh MEC-1 cells after mixing at different effector/target ratios (Figure S8B).

Overall, these findings indicate that enhanced expression of *miR-181b* in CLL cells promotes activation and CTL maturation of T cells via direct B-T interactions.

### 2.5. MiR-181b Affects the Production of IL10 by Regulating c-Fos Expression

**Figure 6:**
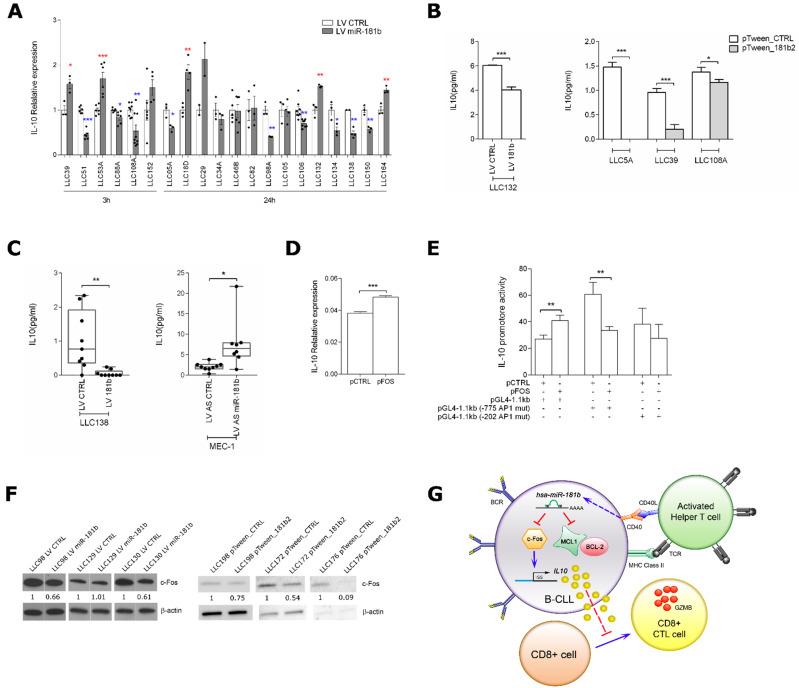
*MiR-181b* targets c-Fos in CLL cells. (**A**) Relative expression of IL10 by RT-qPCR in CLL cells after infection with either LV miR-181b or LV CTRL; *IL10* expression was normalized to the endogenous reference *ACTB*. Data are means ± SEM and technical replicates are shown for each sample as black dot (●). (**B**) ELISA determination of IL10 in the culture medium from CLL cells infected with LV miR-181b or LV CTRL, or transfected with pTween_181b2 or pTween_CTRL and activated on HeLa-CD40L cells. For LLC132 sample, IL10 was measured after three days of activation on HeLa-CD40L cells followed by eight days of culturing without HeLa cells; for LLC5A, LLC39, and LLC108A samples, IL10 was measured after seven days of activation on HeLa-CD40L cells. In the culture medium from HeLa-CD40L cells alone, the IL10 was undetectable (data not shown). Data are mean ± SEM. (**C**) ELISA determination of IL10 in the culture medium from a CTL assay with autologous (LLC138) or allogenic (MEC-1) T-cells. PBMC from LLC138 patient were cultured in a two-round of CTL assay; MEC-1 cells were mixed with T cells from healthy donor 28 (THD28), as described in the experiment reported in Figure 5D. IL10 release was measured in medium after one (MEC-1) or two (LL138) 8-days cycles following B-T cells mixing. Data are median ± range of experimental triplicates. (**D**) Relative expression of *IL10* (by RT-qPCR) in transfected MEC-1 cells after 24 h with either a c-Fos-expressing plasmid (pFOS) or a control plasmid (pCTRL). Data are mean ± SEM. (**E**) Relative luciferase activity of the IL10 promoter in co-transfected MEC-1 cells with pFOS (or pCTRL) and a PGL4 luciferase reporter vector containing the IL10 promoter region in native form or mutated at one of the two AP-1 transcription factor binding sites. The data were normalized to Renilla luciferase activity. Data are mean ± SEM. (**F**) Western blot of c-Fos in purified CLL cells from six CLL patients infected with either LV miR-181b or LV CTRL, or transfected with either pTween_181b2 or pTween_CTRL. β-actin was used as loading control. Densitometric values normalized to β-actin expression are reported. (**G**) Schematic representation of the proposed *miR-181b* mechanism of action. The enhanced expression of *miR-181b* in CLL cells increases the cytotoxic activity vs. leukemic cells by (i) targeting the anti-apoptotic proteins MCL-1 and BCL-2 [16,17], thus sensitizing CLL cells to the extrinsic apoptosis CTL-mediated; and by (ii) reducing the expression of IL10, which inhibits the production of cytokines that promote the generation of effector CTLs [11,34,35]. *p*-values were calculated by Student’s t test (* *p* < 0.05, ** *p* < 0.01, *** *p* < 0.001); in panel A, blue * was used to denote significant decrease of *IL10* expression, while red * was used to denote significant increase of *IL10* expression.

To further investigate the role of the *miR-181b*, we studied factors that might modulate CTL maturation. We focused on IL10, which is known to have a role in the suppression of cell-mediated immune response [31] and that is generally high in plasma from CLL patients [12]. We, therefore, evaluated *IL10* mRNA expression in purified primary CLL cells overexpressing *miR-181b* (Figure S9A) and found that this miRNA downregulated *IL10***,** at the transcriptional level, in a subset of 9 out of 20 patients (Figure 6A). To assess if the microenvironment influences the axis *miR-181b*/IL10, we measured the IL10 protein levels in primary CLL cells overexpressing *miR-181b* and co-cultured with Hela-CD40L. We find an inverted effect of the miRNA on IL10 in LLC132 and LLC39 CLL cells, suggesting that CD40L cooperates with *miR-181b* to reduce the immunosuppressive cytokine (Figure 6B). We then analyzed the supernatant of CD40L-activated CLL cells infected with either LV miR-181b or LV CTRL and cultured in a two-round CTL assay with autologous T-cells, finding a reduction of IL10 secretion after overexpression of *miR-181b* (Figure 6C, left panel). Next, we analyzed the supernatant of CD40L-activated MEC-1 cells, either *miR-181b* depleted or not. These cells were cultured in a CTL assay with allogenic T-cells or alone. In both cases, IL10 secretion increased with *miR-181b* depletion (Figure 6C right panel, and Figure S10).

As a possible factor linking *miR-181b* to IL10, we tested the transcriptional factor c-Fos, which binds the IL10 promoter enhancing IL10 transcription in T cells [32] and was validated as a direct *miR-181b* target in glioma cells [33]. We confirmed this mechanism in B cells. In MEC-1 cells, c-Fos-expressing plasmid (pFOS) increased both IL10 transcription (Figure 6D) and, specifically, the luciferase activity of a reporter vector containing the inducible region of the IL10 promoter (Figure 6E). Reduced c-Fos protein levels were also observed in primary CLL cells from five of six patients upon enhanced expression of *miR-181b* (Figure 6F). To investigate whether *miR-181b* undergoes any regulatory feedback mechanism, we checked the miR expression in six CLL patients either overexpressing c-Fos or not. Among these, four CLL patients showed the decrease of *miR-181b* expression after c-Fos overexpression, suggesting a regulative feedback that could in part explain the downregulation of *miR-181b* in CLL. In two patients, we saw an increase of the miR after c-Fos overexpression, which still indicates a mechanism of regulation c-Fos-mediated, but specific associated factors and/or environmental conditions could be necessary to see a negative regulation of the *miR-181b* by c-Fos (Figure S11).

Taken together, these data suggest that the increased CTL maturation-*miR-181b* mediated occurs through the indirect inhibition of IL10 by this miRNA (Figure 6G).

### 2.6. The In Vivo Model Shows Induced Death of miR-181b-Expressing MEC-1 Cells Only in Presence of Engrafted T Cells

**Figure 7:**
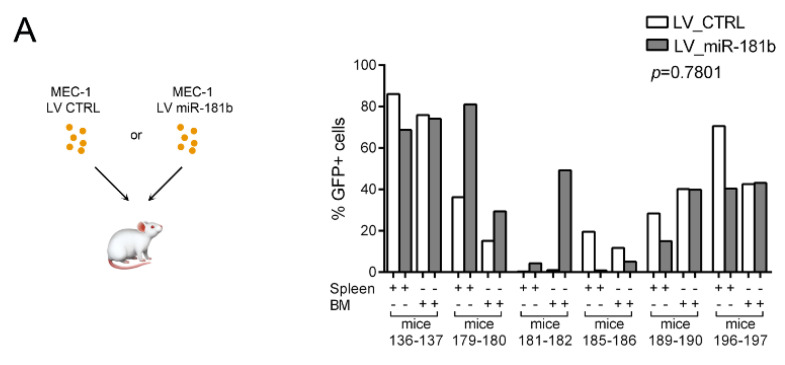

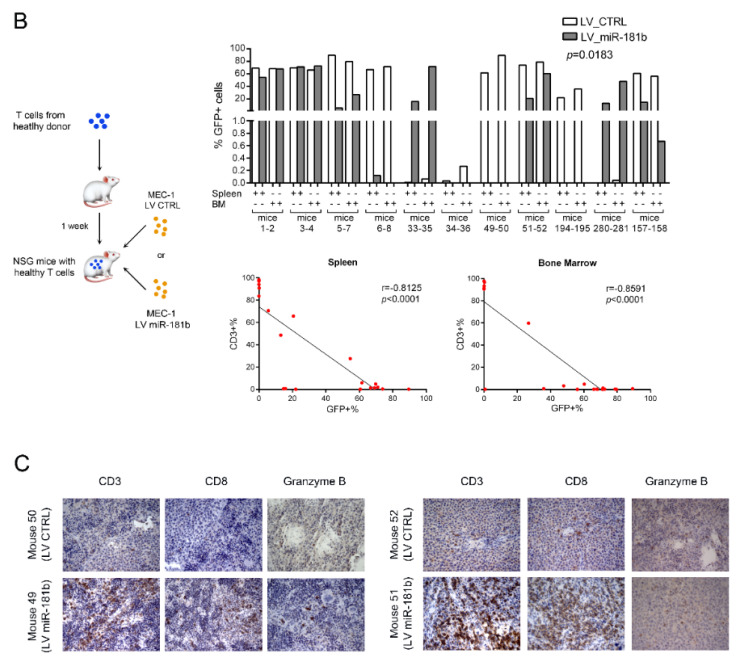
*MiR-181b* induces death of MEC-1 cells in mice with functional T cells. (**A**) Percentages of GFP+ cells on CD45+ cells in spleen and bone marrow of six pairs of NOD.Cg-Prkdcscid Il2rgtm1Wjl/SzJ (NSG) mice injected (i.v.) with 10^7^ infected MEC-1 cells with lentiviral particles encoding GFP alone or with *miR-181b*. (**B**) (Upper) Percentages of GFP+ MEC-1 cells on CD45+ cells in spleen and bone marrow of 11 pairs of NSG mice. Mice were inoculated with either purified 10^6^ T cells from healthy donors or 10^5^ CD34+ cells (57% purity) from human cord blood (mice 157 and 158) and re-inoculated with transduced MEC-1 after one week or six months, respectively. *p*-values were calculated by two-way ANOVA. (Lower) Correlation between the percentage of GFP+ (reported in Figure 6B) and CD3+ cells in spleen and bone marrow of mice; statistical significance was assessed by Pearson’ correlation. Flow cytometry gating strategies used in the experiments are reported in Figure S12. (**C**) Immunohistochemical analysis for CD3, CD8, and granzyme B on spleen sections from two pairs of mice. Pictures were acquired with a Leica microscope using Leica application suite V 4.8.0 software (400X magnification).

To assess the role of *miR-181b* in the T-cell mediated immune response against leukemic cells in vivo, we generated murine models of human CLL using immune-deficient NOD. Cg-Prkdc^scid^ Il2rg^tm1Wjl^/SzJ mice, which do not have functional T cells. MEC-1 cells were infected with either LV miR 181b or LV CTRL and inoculated into six pairs of sibling mice. After four weeks, the percentages of GFP^+^ cells in the bone marrows and spleens of the sacrificed mice were variable, regardless of *miR-181b* (Figure 7A). We performed a similar experiment using 13 pairs of sibling mice in which the immune system was partially restored by inoculation of human T cells from healthy donors (12 pairs) or CD34^+^ cells isolated from human cord blood (one pair). In 2 of the 13 mice pairs that received human T cells, subsequent inoculation of MEC-1 cells did not lead to detectable GFP^+^ cells in the spleen or bone marrow; therefore, these mice were excluded from the analysis. Six of the remaining 10 mice pairs that received *miR-181b*, showed lower GFP^+^ cells and higher CD3^+^ cells in spleens and bone marrows compared to the controls (Figure 7B). Overall, we identified an inverse correlation between GFP^+^ cells and CD3^+^ cells both in the spleens and in the bone marrows (Figure 7B lower). Higher expression of CD3, CD8, and granzyme B was confirmed by immunohistochemistry in two pairs of spleen sections from mice that received LV miR-181b MEC-1 cells (Figure 7C).

## 3. Discussion

Enhancing the immune response is a promising strategy for cancer treatment [36,37]. In this study, we demonstrate the contribution of *miR-181b*, a miRNA extremely down-regulated in CLL and CLL progression [15,17], in the T cells cytotoxic activity. Expression of *miR-181b* is increased at the transcriptional level in primary CLL cells by exogenous activated CD4^+^ T cells through CD40-CD40L interactions. In this context, *miR-181b* facilitates the maturation of cytotoxic T cells and enhances their activity against CLL cells. We also demonstrate that *pri-miR-181a2* levels increase in CLL cells upon contact with activated T cells, while the mature form weakly follows the same trend, likely due to defective processing machinery, already described in CLL [38]. The levels of *miR-130a*, but not of its pri-miRNA, also significantly increase, suggesting that several mechanisms modulate the expression of this gene.

We show that activated both allogenic and autologous T cells increase *miR-181b* expression in CLL upon direct contact, despite the defects reported in the CLL environment [5,39]. Although CD40–CD40L interactions likely occur in lymph nodes of CLL patients [40,41], they might not be sufficiently strong to induce consistent cytoplasmic *miR-181b* expression in CLL cells. Indeed, we observed weak cytoplasmic *miR-181b* staining in the majority of cells in lymph nodes from five CLL patients, while strong or faint nuclear staining for the *pre-miR181b2* and/or *miR-181b* was found in three and two cases, respectively. Based on the experiments performed, we cannot rule out cross-hybridizations of the miRNA with the pre-miRNA probes; however, cytoplasmic signals were almost absent in CLL lymph nodes but were present in the normal tonsils. Overall, these data suggest that impaired processing or transcription of the *miR-181b* contribute to weakening its physiologic role on its targets.

CLL cells do not undergo apoptosis, despite the fact that their BCRs are constitutively activated [9]. This condition should activate CD40 signaling during B-T cell interactions, leading B cells to death. We speculate that the resistance of the CLL cell to death could be due, at least in part, to low *miR-181b* expression. Interestingly, we found that CD40L stimulation of miR-181b-depleted MEC-1 cells resulted in a low yield of CTLs with reduced ability to induce apoptosis. We explain this phenomenon through two mechanisms: (i) Exogenous *miR-181b*, by targeting the anti-apoptotic proteins MCL-1 and BCL-2, sensitizes CLL cells to the extrinsic apoptosis through CD8^+^ T cells; and (ii) *miR-181b* reduces the expression of IL10, which inhibits the production of cytokines that promote the generation of effector CTLs [11,34,35] (Figure 6G).

Concerning the regulation of IL10 mediated by *miR-181b*, we observe a consistent increase of the IL10 protein level upon depletion of *miR-181b* in MEC-1 cells. Increasing the *miR-181b* expression in primary CLL cells determines a heterogeneous response at the IL10 transcriptional level, but a homogeneous decreasing of the secreted cytokine upon CD40L stimulation.

The IL10 transcription modulation by *miR-181b* sustains IL10 as a downstream effector of *miR-181b*. Indeed, the heterogeneity of cells among patients and within individual patient [42] as well as the numerous regulatory feedbacks, could explain the variability on the IL10 transcriptional level as attempt to reestablish *miR-181b*/IL10 cellular equilibrium.

In vitro results are strongly supported by in vivo experiments in that enhanced expression of *miR-181b* in MEC-1 cells sensitized cells to death only in an environment hosting healthy mature T cells. In our mouse model, we mixed allogenic lymphocytes, but the therapeutic role of *miR-181b* in an autologous system of CLL has already been reported [18]. Regarding the T-B cells interaction in CLL, it has been demonstrated that autologous T cells support the proliferation of cognate CLL cells [10,43]. However, when T cells from CLL patients are exogenously activated by CD3/CD28 beads or chimeric antigen receptor (CAR) [44], they trigger a cascade of molecular events that culminate in leukemic cell death. These apparent controversial findings could be explained by evidence that in the CLL environment T cell activation fails to trigger an effective anti-tumor immune response. According to our results, *miR-181b* might be one of those factors and could underlie current and promising therapies that promote leukemic cell death by enhancing interaction between CLL cells and activated T cells [44] (https://clinicaltrials.gov/ct2/show/NCT01013441). Stimulation of CLL cells by activated T cells may not always increase *miR-181b* expression, suggesting the existence of a subset of refractory patients with different cytotoxic T cell response that may be influenced also by the patient genetic trait [45]. So far, we do not have data to observe whether the mechanism we discovered would work irrespective of the genetic alteration common in CLL, and this point will be investigated in the near future. However, a direct modulation of *miR-181b* in CLL cells, concomitant with T cell activation or CD40-CLL stimulation, could be therapeutically effective for the above-described refractory patients and a valid alternative for the management of all CLL patients.

## 4. Materials and Methods

### 4.1. Patients and Healthy Blood Donors

CLL specimens (blood and lymph nodes) and control non-neoplastic tonsil tissues were provided by Policlinico Agostino Gemelli (Rome). Blood from healthy donors was collected at the SS. Annunziata Hospital (Chieti). The characteristics of the CLL patients studied are reported in Table S1. Patients and controls gave written informed consent for the use of their biological samples. Sample collection was approved by the Ethical Committee of the Fondazione Policlinico Agostino Gemelli and by the Health Direction of the SS. Annunziata University Hospital.

### 4.2. Cell Lines and Cultures

HeLa cells expressing CD40 ligand (CD154) (HeLa-CD40L or HeLa-CD154) were previously described [28] and kindly provided from Dr. Laura Rassenti (Moores UCSD Cancer Center); MEC-1 cells were acquired from Leibniz Institute DSMZ-German Collection of Microorganisms and Cell Cultures GmbH (ACC 497); HEK293T cells were acquired from ATCC (CRL-3216 ™) and authenticated by us by short tandem repeat analysis using the PowerPlex16HS kit (Promega, Fitchburg, WI, USA). HeLa-CD40L and MEC-1 cells were cultured in RPMI1640 (Sigma-Aldrich, Saint Louis, MO, USA), HEK293T cells in DMEM (Sigma-Aldrich, Saint Louis, MO, USA). All cultures were maintained at 37 °C in a 5% CO_2_ incubator and the media were supplemented with 10% fetal bovine serum, 1% Pen/Strep, and 1% L-glutamine (Sigma-Aldrich, Saint Louis, MO, USA).

### 4.3. Primary Cell Purification

Peripheral blood mononuclear cells were isolated from whole blood by density gradient centrifugation using Ficoll-Paque PLUS (GE Healthcare, Chicago, IL, USA). CD4+, CD8+, or CD3+ T cells were isolated from PBMCs using the CD4+ T Cell, CD8+ T Cell, or Pan T Cell isolation kits (Miltenyi Biotec, Bergisch Gladbach, Germany). CLL cells were obtained from PBMCs, using the B-CLL Cell Isolation Kit (Miltenyi Biotec, Bergisch Gladbach, Germany), or from whole blood, using the RosetteSep Human B Cell Enrichment Cocktail (Stemcell Technologies, Vancouver, Canada). CD34+ cells were enriched from umbilical cord blood using the CD34 Microbead Kit (Miltenyi Biotec, Bergisch Gladbach, Germany).

### 4.4. Co-Culture of CD4+ T Cells and CLL Cells

CD4+ T cells from healthy donors were seeded in RPMI1640 medium (5 × 10^6^ cells/well) and activated for 3 days using the human T Cell Activation/Expansion Kit (Miltenyi Biotec, Bergisch Gladbach, Germany). Activation of CD4+ T cells was assessed by CD40L expression using flow cytometry (data not shown). Purified CLL cells were co-cultured at 1:1 ratio with CD4+ T cells for 4 and 24 h, then CLL cells were isolated and RNA was analyzed for gene expression. The purity of isolated CLL cells was verified by flow cytometry, which showed an average of 98.9% (co-culture with not-activated T cells) and 98.7% (co-culture with activated T cells). Monoclonal anti-human CD40L antibody was added to the T cell cultures 24 h before T-B cell contact to inhibit interactions; normal mouse IgG was used as the negative control (Table S2).

### 4.5. Activation of PBMC from CLL Patients

PBMCs from CLL patients were seeded in X-VIVO medium (Lonza, Basel, Switzerland) containing 5% human serum (BioWhittaker, Lonza, Basel, Switzerland) at the final concentration of 3.5 × 10^6^ T-cells/mL, activated for 3 days using the human T Cell Activation/Expansion Kit (Miltenyi Biotec, Bergisch Gladbach, Germany) and cultured for additional 24 or 48 h. Then, CLL cells were isolated and RNA was analyzed for gene expression.

### 4.6. RNA Isolation and Reverse Transcription Quantitative PCR Assays

Total RNA was extracted using QIAzol Lysis Reagent (Qiagen, Hilden, Germany), quantified by spectrophotometry on a Nanodrop 2000 instrument (Thermo Scientific, Waltham, MA, USA) and assessed for quality by the RNA HighSens Analysis Kit in the Experion Automated Electrophoresis System (Bio-Rad, Hercules, CA, USA). For pri-miRNA analysis, total RNA was treated with DNase (Roche, Basel, Switzerland).

Total RNA was reverse transcribed using the High-Capacity cDNA Reverse Transcription Kit (Thermo Fisher Scientific, Waltham, MA, USA) and quantitative PCR was performed using the FastStart Universal Probe Mastermix kit (Roche, Basel, Switzerland) or the QuantiFast SYBR Green PCR kit (Qiagen, Hilden, Germany) for IL10. Fragments were amplified using the CFX96 Touch Real-Time PCR System (Bio-Rad, Hercules, CA, USA). Primer sequences and FAM probes were designed using UPL Assay Design Center Roche Software (Table S3). Gene expression was normalized to the endogenous reference genes *RNU44*, *ACTB*, or *GAPDH* as appropriate by the 2^−Δct^ method.

### 4.7. In Situ Hybridization

In situ hybridization (ISH) was performed on formalin-fixed, paraffin-embedded, 4 µm-thick serial sections mounted on positively charged slides from lymph nodes of CLL patients and tonsils of non-cancer patients. Histological sections were deparaffinized, hydrated, washed in PBS, incubated with proteinase K (Dako, Agilent, Santa Clara, CA, USA), washed again in PBS, dehydrated, and incubated overnight at 42 °C with miRCURY LNA detection probes (Table S2). After incubation, slides were washed in 0.5X Saline-Sodium Citrate buffer (SSC) 5 min at room temperature, in 0.5X SSC 5 min at 42 °C and in PBS 1 min. Then, the sections were incubated with Blocking Solution containing maleic acids (Roche, Basel, Switzerland) for 15 min at room temperature and with anti-digoxigenin-AP antibody (Table S2) for 60 min at room temperature. Excess antibodies were removed with 3 min washes in PBS/0.1% Tween. Staining was revealed after incubation minutes in 1X Detection Buffer at 30° C for 30–60. Finally, the slides were washed in deionized water and mounted in aqueous Permanent Mounting Media (Scytek Laboratories, Logan, UT, USA). Images were acquired with an Olympus BX53 microscope.

### 4.8. Nuclear Extraction

Nuclear extraction from CLL cells was performed using the NE-PER Nuclear and Cytoplasmic Extraction kit (Thermo Fisher Scientific, Waltham, MA, USA). Nuclear fractions were dissolved in QIAzol Lysis Reagent (Qiagen, Hilden, Germany) for RNA extraction and gene expression analyses.

### 4.9. Flow Cytometry

Surface B and T cell markers were assessed by flow cytometry using the antibodies reported in Table S2. Cytotoxicity evaluation was performed using Annexin-V antibody (Enzo Life Science, Farmingdale, NY, USA). Cytoplasmic staining with anti-granzyme B was performed as in Lanuti et al., 2012 [46], with some modifications. Briefly, 3–10 × 10^5^ cells were washed in Washing Buffer (WB: PBS1X supplemented with 0.1% sodium azide and 0.5% bovine serum albumin), resuspended in 1 mL of FACS Lysing solution 1X (BD Biosciences, San Jose, CA, USA), mixed and incubated at room temperature in the dark for 10 min, washed in WB, resuspended in 1 mL of permeabilizing solution 2 1X (BD Biosciences, San Jose, CA, USA), and mixed and incubated at room temperature in the dark for 10 min. Then, cells were washed with WB, stained with the GrzB antibody for 30 min at 4 °C in the dark, finally washed in WB, and resuspended in 500 µL of PBS1X.

Data were acquired with a FACSCanto II cell analyzer (BD Biosciences, San Jose, CA, USA) and analyzed with FlowJo v10.0.7 and/or FCS Express 5 software (De Novo Software, Pasadena, CA, USA).

### 4.10. Plasmid Constructs

A 94 bp fragment, including *miR-181b2*, was amplified by PCR from HEK293T cells using specific primers (Table S1). The PCR product was digested with XbaI and XhoI restriction enzymes and cloned into the self-inactivating lentiviral vector pRRLSIN.cPPT.PGK.WPRE (pTween) (gift from Didier Trono; Addgene plasmid #12252). The phosphoglycerate-kinase (hPGK) promoter was removed via BamHI and EcoRV restriction sites and replaced with the EF1 promoter amplified from the pCDH-CMV-MCS-EF1-copGFP vector (System Biosciences, Palo Alto, CA, USA). This vector was designated pTween_181b2. To generate the control vector (pTween_CTRL), self-complementary oligonucleotides (StLoopSCNew_XbaI-XhoI_5′/StLoopSCNew_XbaI-XhoI_3′; Table S3) encompassing a scramble sequence were chemically synthesized (Integrated DNA Technologies) and directionally cloned into the pTween carrying the EF1 promoter instead of the hPGK promoter. Plasmids pAS miR-181b (product # MZIP 181b-PA-1) and pCTRL (PSIH1-H1-siLUC–copGFP; product # LV500A-1) were purchased from System Biosciences.

### 4.11. Lentivirus Packages

To generate the lentivirus expressing *miR-181b* or the control, HEK293T cells were co-transfected with the envelope plasmid pMD2.G, the packaging plasmid pCMVR8.74 (gift from Didier Trono; Addgene plasmids #12259 and #22036), and either pTween_181b2 or pTween_CTRL using the Calcium Phosphate Transfection Kit (Thermo Fisher Scientific, Waltham, MA, USA). After 48 h, the culture supernatants were harvested, cleared by centrifugation at 1500 rpm at room temperature for 10 min, and filtered through a 0.45 µm syringe filter. The obtained lentiviruses were named LV miR-181b and LV CTRL.

Lentiviruses expressing miR-181b antisense (AS) sequences, or the control, were generated using pAS miR-181b or pCTRL and the pPACKH1 HIV Lentivector Packaging Kit (System Biosciences, Palo Alto, CA, USA). The obtained lentiviruses were named LV AS miR-181b and LV AS CTRL. Virus titers were assessed by the percentage of GFP-positive cells using flow cytometry, according to the formula: Viral particles/µL = (no. of cells) × (% GFP-positive cells)/volume of virus solution.

### 4.12. Cell Infection

MEC-1 cells were infected with lentiviruses at 10 multiplicity of infection (MOI), while CLL cells or PBMCs were infected at MOI 0.5. Briefly, cells were suspended in medium containing lentivirus and polybrene (10 μg/mL), seeded in 6-well plates, and centrifuged for 45 min at 1800 rpm. After 24 h, the virus-containing medium was replaced with a complete culture medium. Transduction efficiency was determined from the percentage of GFP-positive cells using flow cytometry or by measuring the transducer gene miR-181b using RT-qPCR.

### 4.13. Generation of Effectors CTLs

Effector cytotoxic lymphocytes (CTLs) were generated as described by Chu et al. [28], with some modifications. MEC-1 cells infected with either LV AS miR181b or LV AS CTRL were seeded on HeLa-CD40L (40:1, MEC-1/HeLa-CD40L) for 72 h in AIM-V (Thermo Fisher Scientific, Waltham, MA, USA) supplemented with 2.5% FBS (Sigma-Aldrich, Saint Louis, MO, USA) and recombinant human IL4 (10 ng/mL, BD Biosciences, San Jose, CA, USA). CD40-activated MEC-1 cells were placed in a new well to allow any contaminant HeLa cells to attach. After 3 h, floating cells were collected, treated with mitomycin C (80 µg/mL, Sigma-Aldrich, Saint Louis, MO, USA) for 1 h at 37 °C, and then washed 3 times with serum-free AIM-V. Cells were seeded at 0.5–2.3 × 10^5^/well and co-cultured with purified CD3+ T cells from healthy donors at an effector/target (E/T) ratio of 2:1 in serum-free AIM-V. After 4 days of B and T lymphocytes co-culture, cells were supplemented with IL-2 (50 U/mL, BD Biosciences, San Jose, CA, USA) and cultured for additional 4 days. On day 8, T cells were harvested and analyzed by flow cytometry, used as effector cells in CTL assays, or mixed with CLL cells for the second round of CTL generation.

### 4.14. CTL Assay

CD8+ T cells from healthy donors were mixed with infected MEC-1 (1 × 10^4^) with either LV miR-181b or LV CTRL at the E/T ratios 1:1, 5:1, 10:1. After 4 or 7 h, MEC-1 cells were labeled with Annexin-V and analyzed by flow cytometry (Figure S7).

T cells from CTL generation assay were mixed with fresh MEC-1 cells (0.5–30 × 10^4^) pre-labeled with Violet Proliferation Dye 450 (VPD, BD Biosciences, San Jose, CA, USA) in AIM-V medium at different E/T ratios for 1 or 2 h at 37° C. After co-culture, isolated MEC-1 cells were stained with Annexin V and analyzed by flow cytometry (Figure 5D and Figure S8B).

### 4.15. Transwell Assays

MEC-1 cells infected with LV miR-181b or LV CTRL were seeded at 1.8 × 10^5^/well. Cells were co-cultured into the upper chamber of transwell dishes (0.4 µm pore size; Corning) with or without activated T cells from healthy donor 22 (E/T ratio, 1:9) in RPMI medium supplemented with 20% FBS. MEC-1 cells infected with either LV AS miR181b or LV AS CTRL were stimulated on HeLa-CD40L for 3 days and co-cultured into the upper chamber of transwell dishes with or without activated T cells from healthy donor 22 (E/T ratio, 2:1), as in the generation of effector CTLs. Activated and non-activated T cells in the upper and lower chambers, respectively, were from the same healthy donor. T cells in the lower chamber were cultured at the same E/T ratio as in the upper chamber. After 8 days of incubation, the T cells in the lower chamber were analyzed by flow cytometry.

### 4.16. IL10 ELISA

IL10 was measured in the culture medium using the Human IL-10 ELISA Ready-SET-Go kit (Affymetrix, Santa Clara, CA, USA). Primary CLL cells overexpressing *miR-181b*, or MEC-1 silencing *miR-181b*, and their relative controls, were activated on HeLa-CD40L cells for 3 days, then cells were cultured at different conditions (alone or in a CTL assay) and the supernatant was assayed for the presence of IL10.

### 4.17. Cell Transfection

Transient transfections of plasmids (2 µg for 1 × 10^6^ cells), miRNA mimics (50 pmol for 1 x10^6^ cells) or siRNA (50 pmol for 1 × 10^6^ cells) in MEC-1 cells were performed using the U-016 program of the Amaxa Nucleofector II (Lonza, Basel, Switzerland). Transient transfections of plasmids (2µg for 3–5 × 10^6^ cells) in primary CLL cells were performed using the U-016 program of the Amaxa Nucleofector II (Lonza, Basel, Switzerland).

### 4.18. Dual-Luciferase Reporter Assay

IL10 promoter activity was measured in MEC-1 cells. FOS expression vector pLX304-FOS-V5 (pFOS) (gift from William Hahn; Addgene plasmid #59140) [47] or control plasmid pLX304 (pCTRL) (gift from by David Root; Addgene plasmid # 25890) [48] were co-transfected with a reporter vector carrying the native IL10 promoter upstream to the luciferase gene (PGL4-wt) or a mutated IL10 promoter in one of the two AP-1 binding sites (PGL4-mut_775 or PGL4 mut_202) [32]. As an internal control, MEC-1 cells were additionally transfected with a Renilla luciferase reporter vector (pRL-TK; Promega, Madison, WI, USA). After 24 h, luminescence was evaluated with the Dual-Glo Luciferase Assay System (Promega, Madison, WI, USA) by a Lumat LB 9507 luminometer (Berthold Technologies, Bad Wildbad, Germany).

### 4.19. Western Blotting

Cells were collected and lysed in cold protein lysis buffer (50 mM Tris-HCl, 150 mM NaCl, 1% Igepal CA-630, 1 mM EDTA, Sigma-Aldrich, Saint Louis, MO, USA) supplemented with Protease and Phosphatase Inhibitors Cocktail 3 (Sigma-Aldrich, Saint Louis, MO, USA). Cells lysates were run on Criterion TGX Stain-Free pre-cast gels (Bio-rad, Hercules, CA, USA) and blotted on Immun-Blot PVDF membranes (Bio-rad, Hercules, CA, USA). Primary and secondary antibodies are reported in Table S2. All antibodies were diluted in 5% non-fat dry milk. To quantify Western blot signals, high-resolution digital images of autoradiographs were acquired, and band signals were quantified using ImageJ software.

### 4.20. In Vivo Experiments

NOD.Cg-Prkdcscid Il2rgtm1Wjl/SzJ (NSG) mice (Charles River Laboratories, Wilmington, MA, USA) of both sexes were bred and housed under specific pathogen-free conditions in temperature-controlled cages at the institutional animal facility of the G. d’Annunzio University. Animal experiments followed the regulations of the Italian law and the project, which predated the adoption of the d.lgs. n. 26/2014, was approved by the Interinstitutional Committee on Animal Research and Ethics of the Universities of Chieti-Pescara, Teramo, L’Aquila and the Experimental Zooprophylactic Institute of Abruzzo-Molise (CEISA) (Protocol UNCHD12#222/2014). Four/eight-week-old mice were used. Six sibling pairs of mice were injected intravenously with 10^7^ MEC-1 cells infected with either LV miR-181b or LV CTRL; 10 sibling pairs were injected intravenously with 10^6^ healthy T cells and, after 1 week, with infected MEC-1 cells; one sibling pair was injected with 10^5^ enriched CD34+ cells from human cord blood and, after 6 months, with infected MEC-1 cells. In both experiments, mice were euthanized by CO_2_ inhalation 4 weeks after MEC-1 cells injection. Spleens were homogenized and filtered through 40-µm cell strainers. Bone marrow cells were obtained by flushing the femora, tibiae, and hip bones with PBS and then filtering through 40-µm cell strainers.

### 4.21. Immunohistochemical Analyses

Spleen samples were fixed in 10% buffered formalin and embedded in paraffin; 5 µm sections were stained with hematoxylin and eosin (BioOptica, Milan, Italy). After antigen retrieval at pH 9 for 20 min in a microwave, blank serial sections were incubated with the antibodies reported in Table S2. Bound antibodies were detected using Streptavidin Peroxidase reagent (Thermo Scientific, Waltham, MA, USA) and Liquid DAB+ Substrate Chromogen System (Dako, Agilent, Santa Clara, CA, USA). Images were acquired with a Leica microscope.

### 4.22. Statistical Analyses

Statistical analyses were performed using GraphPad Prism 6.01 (GraphPad Software, San Diego, CA, USA). Normality of distributions data was assessed by the D’Agostino & Pearson test; based on the normality test, unpaired *t*-test or Mann–Whitney U-test and paired t-test or Wilcoxon matched-pairs test were used. Pearson’ coefficient was used to analyze the linear statistical dependency of two variables. Statistical tests were two-sided; *p*-values ≤ 0.05 were considered significant.

### 4.23. Availability of Data

All the raw data are freely available at the code-hosting platform GitHub (https://github.com/VeroneseVisoneLabs).

## 5. Conclusions

We demonstrate that the down-regulation of *miR-181b* in CLL cells is involved in the immune dysfunction that characterizes this disease. Restoration of the physiological *miR-181b* activity in CLL cells may be a challenging novel approach to treat CLL patients.

## Data Availability

The data presented in this study are openly available at the code-hosting platform GitHub (https://github.com/VeroneseVisoneLabs).

## Supplementary Materials

The following are available online at https://www.mdpi.com/2072-6694/13/2/257/s1; Table S1: Characteristics of CLL patients included in the study. NA: no data available. Table S2. Fluorochrome-conjugated reagents, antibodies and labeled probes used in the study. Table S3. Primers for reverse transcription (RT), quantitative PCR (qPCR) and cloning. Figure S1. Evaluation of CLL cells activation. Figure S2. Activated CD4+ T cells from CLL patients retain the ability to increase the expression of *miR-181b* in CLL cells. Figure S3. Anti-CD40L mAb blocks the CD40L signaling. Figure S4. *MiR-181b* expression in whole cell and nuclear compartments of B cells from CLL patients. Figure S5. T cells staining in section from lymph node of CLL patients. Figure S6. Controls of *miR-181b* expression and of its activity after ectopic transduction of LV miR-181b or LV AS miR-181b by lentiviruses technology. Figure S7. *MiR-181b* promotes the death of leukemia cells. Figure S8. Depletion of *miR-181b* from B cells reduces T cell activation and maturation. Figure S9. Evaluation of CLL cells infection. Figure S10. Depletion of *miR-181b* in CLL cell line increases IL10 secretion. Figure S11. C-Fos protein regulates *miR-181b* expression. Figure S12. Flow cytometry gating strategy in in vivo experiments. Full unedited blot of figure S6. Full unedited blot of Figure 6E. Full unedited blot of Figure S11.
