# Supplementary material for: Enhanced Expression of miR-181b in B Cells of CLL Improves the Anti-Tumor Cytotoxic T Cell Response
Source: Cancers (Basel). 2021 Jan 12;13(2):257. doi: 10.3390/cancers13020257 (PMC7826592; doi:10.3390/cancers13020257)
Supplement: Supplementary file 1 [file cancers-13-00257-s001.pdf]

cancers

Supplementary materials

Enhanced Expression of *miR-181b* in B Cells of CLL Improves the Anti-Tumor Cytotoxic T Cell Response

Mirco Di Marco [1,2,†], Serena Veschi [1,2,†], Paola Lanuti [1,3], Alice Ramassone [1,2], Stefania Pacillo [1,2], Sara Pagotto [1,2], Felice Pepe [1,2], Jonahunnatha Nesson George-William [1,2], Claudia Curcio [1,3], Marco Marchisio [1,3], Sebastiano Miscia [3], Idanna Innocenti [4], Francesco Autore [4], Barbara Vannata [4], Patrizia Di Gregorio [5], Mario Di Gioacchino [1,3], Silvia Valentinuzzi [1,2], Manuela Iezzi [1,3], Renato Mariani-Costantini [1,2], Luigi Maria Larocca [6], Luca Laurenti [4], Angelo Veronese [1,2] and Rosa Visone [1,2,*]

1 Center for Advanced Studies and Technology (CAST), G. d'Annunzio University, 66100 Chieti, Italy; dma-mirco@gmail.com (M.D.M.); veschi@unich.it (S.V.); p.lanuti@unich.it (P.L.); alice.ramassone@unich.it (A.R.); stefania.pacillo28@gmail.com (S.P.); sara.pagotto@unich.it (S.P.); felixpepe@hotmail.it (F.P.); jonahnesson@gmail.com (J.N.G.-W.); claudia.curcio@unito.it (C.C.); m.marchisio@unich.it (M.M.); m.digioacchino@unich.it (M.D.G.); valentinuzzis@gmail.com (S.V.); miezzi@unich.it (M.I.); rmc@unich.it (R.M.-C.); a.veronese@unich.it (A.V.)
2 Department of Medical, Oral and Biotechnological Sciences, G. d'Annunzio University, 66100 Chieti, Italy
3 Department of Medicine and Aging Sciences, G. d'Annunzio University, 66100 Chieti, Italy; sebastiano.miscia@unich.it
4 Fondazione Policlinico Universitario A. Gemelli IRCCS, 00168 Rome, Italy; idanna.innocenti@yahoo.it (I.I.); francesco_autore@yahoo.it (F.A.); bvannata@hotmail.it (B.V.); Luca.Laurenti@unicatt.it (L.L.)
5 Institute of Transfusion Medicine, "Ss. Annunziata" Hospital, 66100 Chieti, Italy; patrizia.digregorio@asl2abruzzo.it
6 Institute of Pathological Anatomy, Fondazione Policlinico Universitario A. Gemelli IRCCS t, 00168 Rome, Italy; luigimaria.larocca@unicatt.it (L.M.L.)
* Correspondence: r.visone@unich.it; Tel.: +39-0871-541498
† These authors contributed equally to this work.

Citation: Di Marco, M.; Veschi, S.; Lanuti, P.; Ramassone, A.; Pacillo, S.; Pagotto, S.; Pepe, F.; George-William, J.N.; Curcio, C.; Marchisio, M.; et al. Enhanced Expression of *miR-181b* in B Cells of CLL Improves the Anti-Tumor Cytotoxic T Cell Response. *Cancers* **2021**, *13*, 257. https://doi.org/10.3390/cancers13020257

Received: 12 December 2020
Accepted: 08 January 2021
Published: 12 January 2021

Publisher's Note: MDPI stays neutral with regard to jurisdictional claims in published maps and institutional affiliations.

Copyright: © 2021 by the authors. Submitted for possible open access publication under the terms and conditions of the Creative Commons Attribution (CC BY) license (http://creativecommons.org/licenses/by/4.0/).

Supplementary materials

Table S1. Characteristics of CLL patients included in the study. NA: no data available.

ID patients	Sex	Age	IGHV homology	Zap-70	WBC (x10^9/liter)	ALC (x10^9/liter)	Binet/Rai Stage
**LLC3A**	M	82	mutated	negative	80	64	BII
**LLC5A**	M	59	mutated	negative	50	43	BII
**LLC15**	M	44	unmutated	negative	11,5	6,5	A0
**LLC18A**	F	77	mutated	positive	82	71	BII
**LLC18D**	F	78	mutated	positive	87	83	AII
**LLC20A**	M	57	mutated	negative	31,6	27,1	A0
**LLC28**	M	59	mutated	negative	60	55	BII
**LLC29**	M	82	unmutated	positive	29,9	24,1	A0
**LLC34A**	M	64	mutated	negative	24,2	19,6	BII
**LLC34E**	M	68	mutated	negative	33,9	NA	BII
**LLC35**	F	56	mutated	negative	34,3	28,7	BII
**LLC35A**	F	57	mutated	negative	23	20	BII
**LLC39**	M	42	mutated	negative	68	61	BII
**LLC41**	F	68	mutated	negative	40	28,9	BII
**LLC42**	M	60	mutated	negative	92	78	BII
**LLC43**	M	78	NA	positive	57	47	A0
**LLC44**	F	70	NA	positive	42	36	BII
**LLC45A**	F	76	unmutated	negative	60	50	CIV
**LLC45B**	F	78	unmutated	negative	56,6	49,4	BII
**LLC46**	M	65	unmutated	positive	37,2	35	BII
**LLC46B**	M	69	unmutated	positive	17,7	13,3	BII
**LLC47**	F	67	mutated	negative	15,3	8,4	A0
**LLC47A**	F	71	mutated	negative	24,5	18,7	BII
**LLC48**	M	67	unmutated	negative	33,4	28,6	BII
**LLC51**	M	78	mutated	positive	16	13	BII
**LLC52**	M	73	mutated	negative	29,1	25,3	AII
**LLC52A**	M	74	mutated	negative	22,6	19,5	AII
**LLC52B**	M	74	mutated	negative	33	28	BII
**LLC53A**	F	73	mutated	negative	26,5	21,8	A0
**LLC58**	M	63	mutated	negative	86	67,6	AI
**LLC58A**	M	68	mutated	negative	85	79	BII
**LLC59**	F	62	mutated	negative	34,3	25,6	A0
**LLC62**	M	52	unmutated	negative	24,0	NA	A0
**LLC62A**	M	52	unmutated	negative	29,7	23,2	BI
**LLC62B**	M	53	unmutated	negative	49	44	BI
**LLC67-8**	M	74	mutated	positive	86,7	NA	NA
**LLC67-9**	M	70	mutated	positive	NA	NA	NA
**LLC70**	F	69	unmutated	positive	72,7	60,2	BII
**LLC70A**	F	69	unmutated	positive	134	125	BII
**LLC82**	M	52	unmutated	negative	64,7	52	AI
**LLC87**	F	53	mutated	negative	43,4	35	A0
**LLC87A**	F	54	mutated	negative	37,2	32,3	BII
**LLC88A**	M	68	mutated	NA	90	72	BII
**LLC98**	M	78	unmutated	negative	38,7	30	BII
**LLC98A**	M	78	unmutated	negative	82,8	62	BII
**LLC105**	F	87	mutated	negative	52	43,7	BII
**LLC106**	M	67	unmutated	positive	74,8	63,6	BII
**LLC108**	F	62	mutated	positive	52,3	33	BII
**LLC108A**	F	63	mutated	positive	109	90	BII
**LLC129**	F	57	mutated	NA	77	57,8	AI
**LLC130**	M	58	mutated	negative	63,8	53,4	BII
**LLC132**	M	63	mutated	NA	31,1	24,8	A0
**LLC134**	F	72	unmutated	positive	28	20	A0
**LLC138**	F	68	mutated	negative	48,1	41,7	AI
**LLC150**	F	79	mutated	negative	52,3	46,4	AI
**LLC152**	M	75	unmutated	negative	140	102	BII
**LLC164**	M	48	unmutated	negative	49	40	AI
**LLC172**	M	NA	unmutated	NA	65	54	BII
**LLC176**	F	83	NA	NA	23,9	17,3	A0
**LLC197**	F	66	Mutated	NA	128	NA	BII
**LLC198**	M	NA	NA	NA	NA	NA	NA

Table S2. Fluorochrome-conjugated reagents, antibodies and labeled probes used in the study.

Antibody	dilution	Vendor	Cat. No.	Technique
Anti-CD5	1:30	BD Biosciences	555353	FC
Anti-CD19	1:30	BD Biosciences	555414	FC
Anti-CD19	1:30	BD Biosciences	348814	FC
Anti-CD16	1:30	Miltenyi Biotec	130-091-246	FC
Anti-CD45	1:15	BD Biosciences	340910	FC
Anti-CD38	1:30	BD Biosciences	345806	FC
Anti-CD38	1:30	BD Biosciences	563251	FC
Anti-CD3	1:30	Miltenyi Biotec	130-080-401	FC
Anti-CD3	1:30	BD Biosciences	341111	FC
Anti-CD8	1:50	BD Biosciences	555635	FC
Anti-CD8	1:50	BD Biosciences	345775	FC
Anti-CD4	1:30	Miltenyi Biotec	130-091-232	FC
Anti-CD95	1:50	BD Biosciences	340481	FC
Anti-CD34	1:30	Miltenyi Biotec	130-095-393	FC
Anti- CD40L	1:50	Miltenyi Biotec	130-092-289	FC
Anti-Granzyme B	1:30	BD Biosciences	563388	FC
Annex V	1:200	Enzo Life Science	Alx-209-252-T100	FC
VPD 450	1:1000	BD Biosciences	562158	FC
Vibrant Dye Cycle	1:750	Life Technologies	V35003	FC
Anti-human CD40L	1:50	Vinci Biochem	ANC-353-020	T/B
Normal mouse IgG	1:20	SCBT	sc-2025	T/B
Scramble-miR control	1:100	Exiqon	99004-15	ISH
Hsa-pre-miR-181b	1:50	Exiqon	Custom	ISH
Hsa-miR-181b	1:100	Exiqon	38488-15	ISH
Anti-Digoxigenin-AP	1:800	Roche	11093274910	ISH
Anti-human CD3	1:50	Dako	M7254	IHC
Anti-human CD8	1:50	Dako	M7103	IHC
Anti-mouse		Dako	K4001	IHC
Anti-Granzyme B	1:50	Leica Biosystem	NCL-L-GRAN-B	IHC
Anti-FOS	1:200	Invitrogen	38-4950	WB
Anti-β-actin	1:36000	Cell Sgnalling	4967	WB
Anti-BCL2	1:1000	Dako	M0887	WB
Anti-γ-tubulin	1:400	SCBT	sc-7396	WB
Anti-mouse IgG	1:3000	SCBT	sc-2005	WB
Anti-goat IgG	1:3000	SCBT	sc-2020	WB
Anti-rabbit IgG	1:3000	Biorad	1706515	WB

FC: Flow Cytometry; T/B: lymphocytes T B interaction; ISH: In Situ Hybridization; IHC: Immunohistochemical;.WB: Western Blot.

Table S3. Primers for reverse transcription (RT), quantitative PCR (qPCR) and cloning.

Primer name	Sequence	Technique
RT_ U44	GTTGGCTCTGGTGCAGGGTCCGAGGTATTCGCACCAGAGCCAACAG-TCAGTT	RT
RT_181b	GTTGGCTCTGGTGCAGGGTCCGAGGTATTCGCACCAGAGCCAACACCCAC	RT
RNU44_FW	GCGGCGGCCTGGATGATGATAG	qPCR
181b_FW	GCGGCGGAACATTCATTGCTG	qPCR
Universal_RV	GTGCAGGGTCCGAGGT	qPCR
CD95_FW	ATGGCCAATTCTGCCATAAG	qPCR
CD95_RV	TGACTGTGCAGTCCCTAGCTT	qPCR
FOS_FW	ACTACCACTCACCCGCAGAC	qPCR
FOS_RV	CCAGGTCCGTGCAGAAGT	qPCR

IL10_FW	CATCGATTTCTTCCCTGTGA	qPCR
IL10_RV	CGTATCTTCATTGTCATGTAGGC	qPCR
primiR-181b2	TaqMan Pri-miRNA Assays (Applied Biosystem) Hs03303356_pri	RT-qPCR
StLoopSCNewXbaI-XhoI_5′	AATTTCTAGACCGGGCGCGATAGCGCTAATAATTTCACTT-GAAATTATTAGCGCTATCGCGCTTTTTCTCGAGTTAA	Cloning
StLoopSCNew XbaI-XhoI _3′	TTAACTCGAGAAAAAGCGCGATAGCGCTAA-TAATTTCAAGTGAAATTATTAGCGCTATCGCGCCCGGTCTAGAAATT	Cloning
miR-181b2 pos:127455984_FW	AATTTCTAGAAAACACTGATGGCTGCACTC	Cloning
miR-181b2 pos:127456078_RV	AATTGAGCTCTTGTTTGGTCCGCAGTTTGC	Cloning

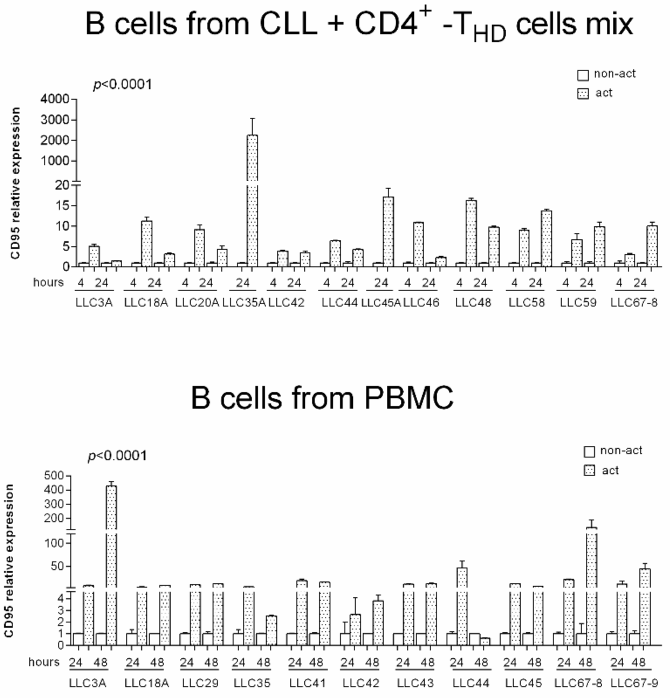

Figure S1. Evaluation of CLL cells activation. CLL cell activation was verified by RT-qPCR of *CD95* in (A) purified CLL cells co-cultured with activated vs. non-activated T cells, and (B) purified CLL cells isolated from activated or non-activated PBMCs from CLL patients. Relative expression values were determined by RT-qPCR; *CD95* data were normalized to the endogenous references ACTB with the $2^{-\Delta ct}$ method. For each patient, the relative expression of *CD95* was normalized to the level of non-activated sample at 4 (A) or 24 (B) hours. Data are presented as mean ± SEM and technical replicates are shown for each sample as black dot. *P*-values were calculated using Wilcoxon test in activated *vs.* non-activated CLL cells.

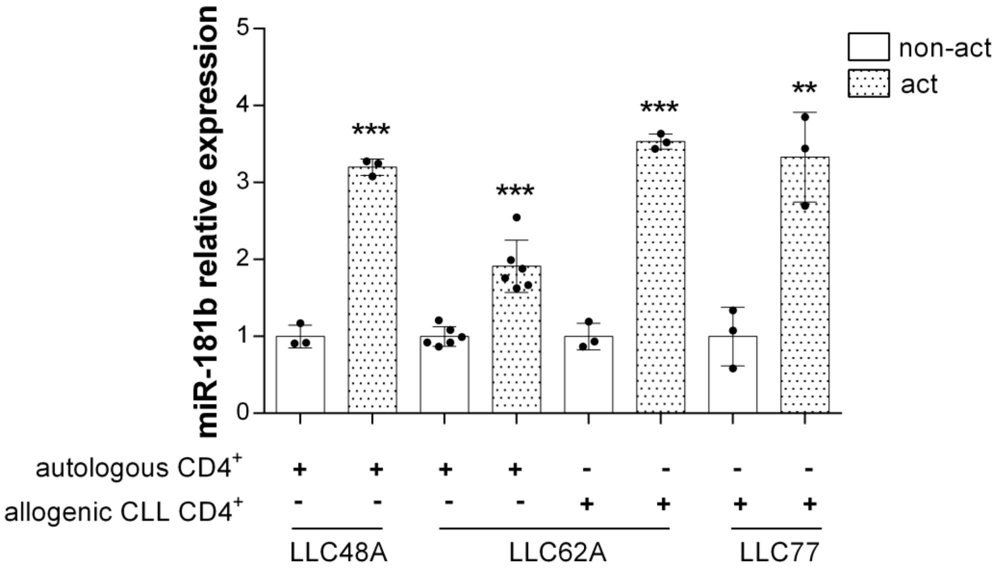

Figure S2. Activated CD4⁺ T cells from CLL patients retain the ability to increase the expression of *miR-181b* in CLL cells. Relative expression (by RT-qPCR) of *miR-181b* in purified CLL cells after 24 h of co-culture with activated or non-activated CD4⁺ T cells, either autologous (LLC48A and LLC62A) or CLL allogenic (LLC62A and LLC77). For each patient, the relative expression of *miR-181b* was normalized to the level of non-activated sample. Data are means ± SEM and technical replicates are shown for each sample as black dot. ** $p < 0.01$, *** $p < 0.001$ by Student's *t* test.

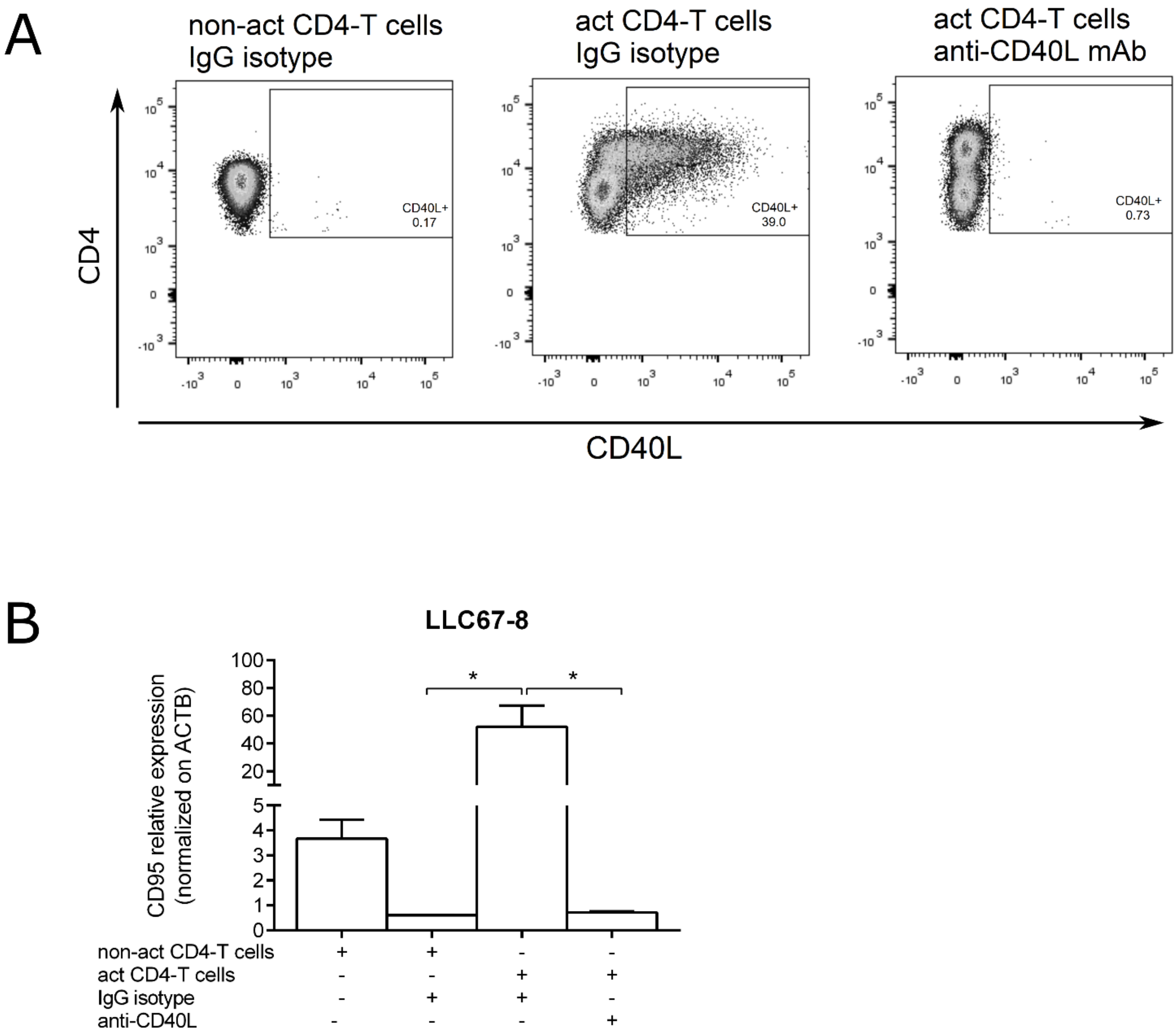

Figure S3. Anti-CD40L mAb blocks the CD40L signaling. A) Percentage of CD4+/CD40L+ cells after activation of T cells from Healthy donor and treatment with anti-CD40L mAb or isotype control. B) Relative expression of CD95 in CLL patients (LLC67-8) showed in Figure 3. Relative expression values were determined by RT-qPCR; *CD95* data were normalized to the endogenous references *ACTB* with the $2^{-\Delta ct}$ method. Data are presented as mean ± SEM. P-values were calculated using Student's t test (*p < 0.05, **p < 0.01 and ***p < 0.001).

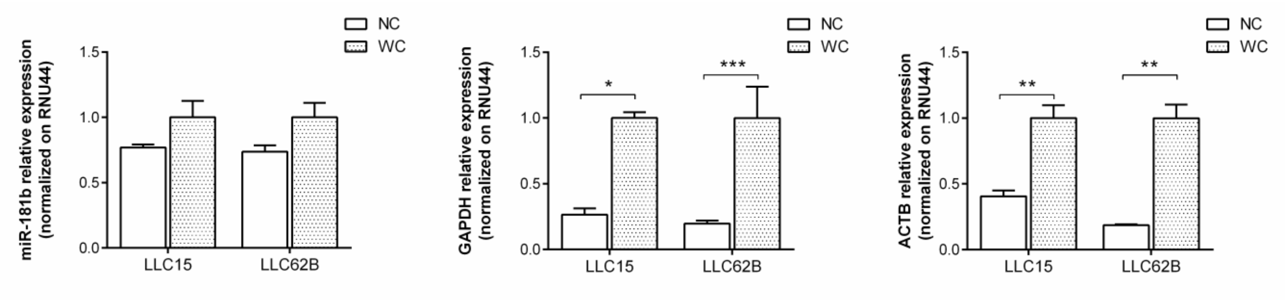

Figure S4. ***MiR-181b*** **expression in whole cell and nuclear compartments of B cells from CLL patients.** Expression levels ($2^{\wedge Ct}$ values) of *miR-181b*, *ACTB* and *GAPDH* in whole cell (WC) and

nuclear (NC) fractions of purified CLL cells from peripheral blood. Data were normalized to the endogenous nuclear reference *RNU44;* then, for each patient, the relative expression of *miR-181b*, *ACTB* and *GAPDH* was normalized to the level of whole cell sample. Data are means ± SEM of technical triplicates. **p* <0.05, ***p*<0.01 and ****p* < 0.001 by Student's *t* test

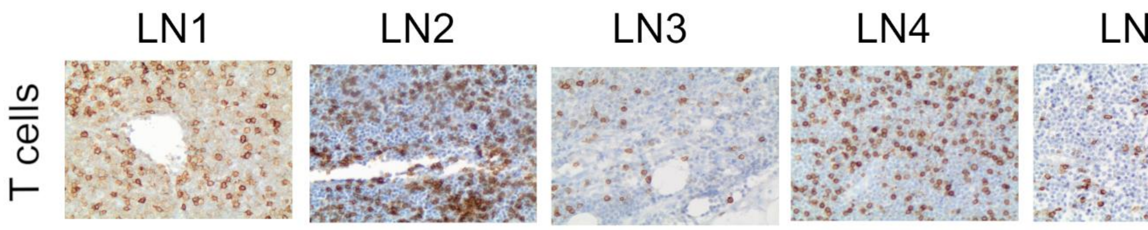

.

Figure S5. T cells staining in section from lymph node of CLL patients. Immunohistochemical analysis for CD5 (LN1) and CD3 (LN2, LN3, LN4, LN5) on lymph node sections from CLL patients, (400X magnification).

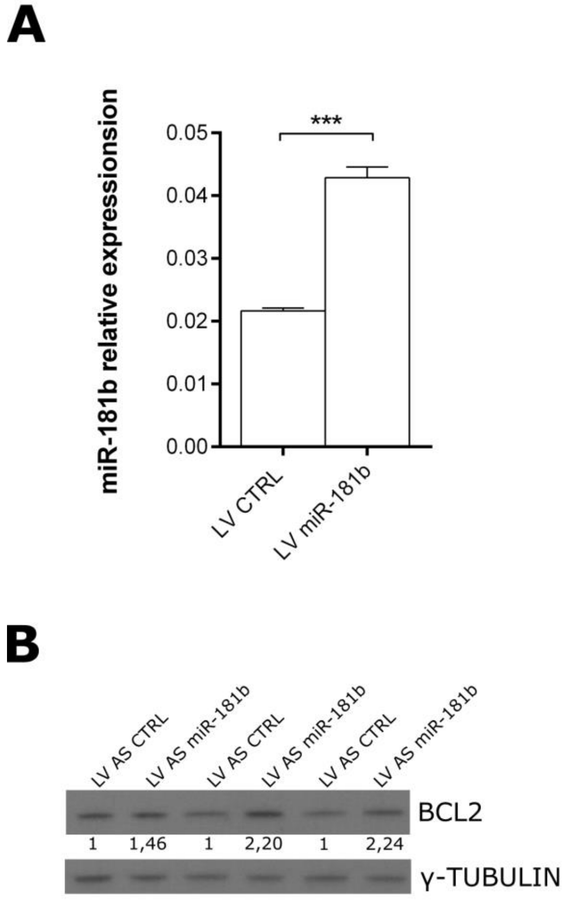

Figure S6. Controls of *miR-181b* expression and of its activity after ectopic transduction of LV *miR-181b* or LV AS *miR-181b* by lentiviruses technology. (A) Expression level of *miR-181b* in MEC-1 cells after 24 hours of infection with LV CTRL or LV miR-181b. Data were normalized to the endogenous reference *RNU44*; data are reported as mean ± SEM of technical triplicates. ***$p < 0.001$ by Student's *t* test. (B) Western blot analysis of BCL2 and γ-TUBULIN in three different transfections of MEC-1 cells with LV AS CTRL or LV AS miR-181b. BCL2 protein levels were normalized to the levels of γ-TUBULIN; then, for each transfection, the relative level of BCL2 was normalized to the level of control sample (LV AS CTRL).

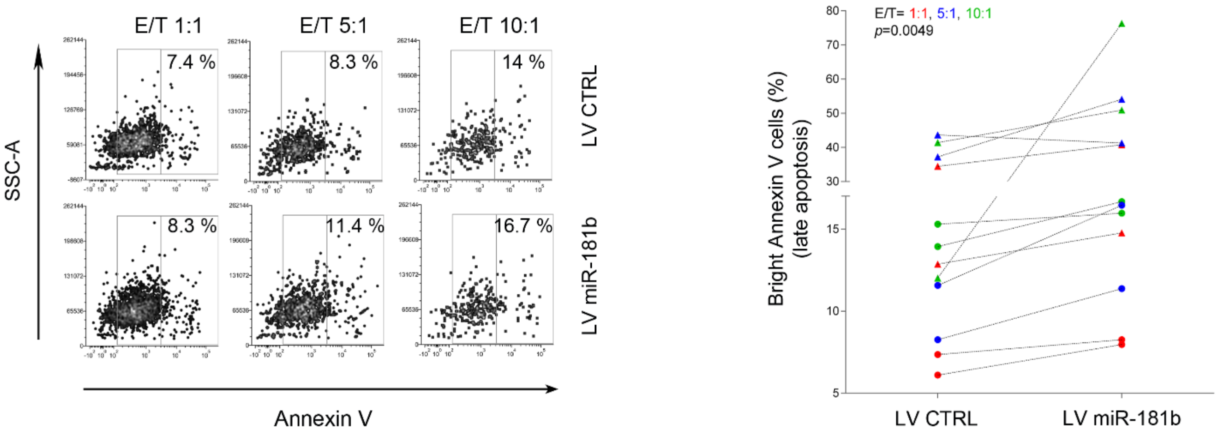

Figure S7. MiR-181b promotes the death of leukemia cells. Percentages of GFP+ /AnnV+ MEC-1 cells infected with either LV CTRL or LV miR-181b. Transduced MEC-1 cells were incubated with effector CD8+ T cells from healthy donors at various effector/target (E/T) ratios. After 4 or 7 hours late apoptosis was evaluated. (Left) Representative plots of four similar independent experiments are shown. (Right) Percentage of AnnV+ cells in late apoptosis (bright annexin) in samples from 4 experiments performed at various E/T ratio, 1:1 (red), 5:1 (blue), 10:1 (green), and at various time points, 4 hours (circles) and 7 hours (triangles). Significance between the two groups (LV CTRL vs LV miR-181b) was determined by Wilcoxon's signed rank test.

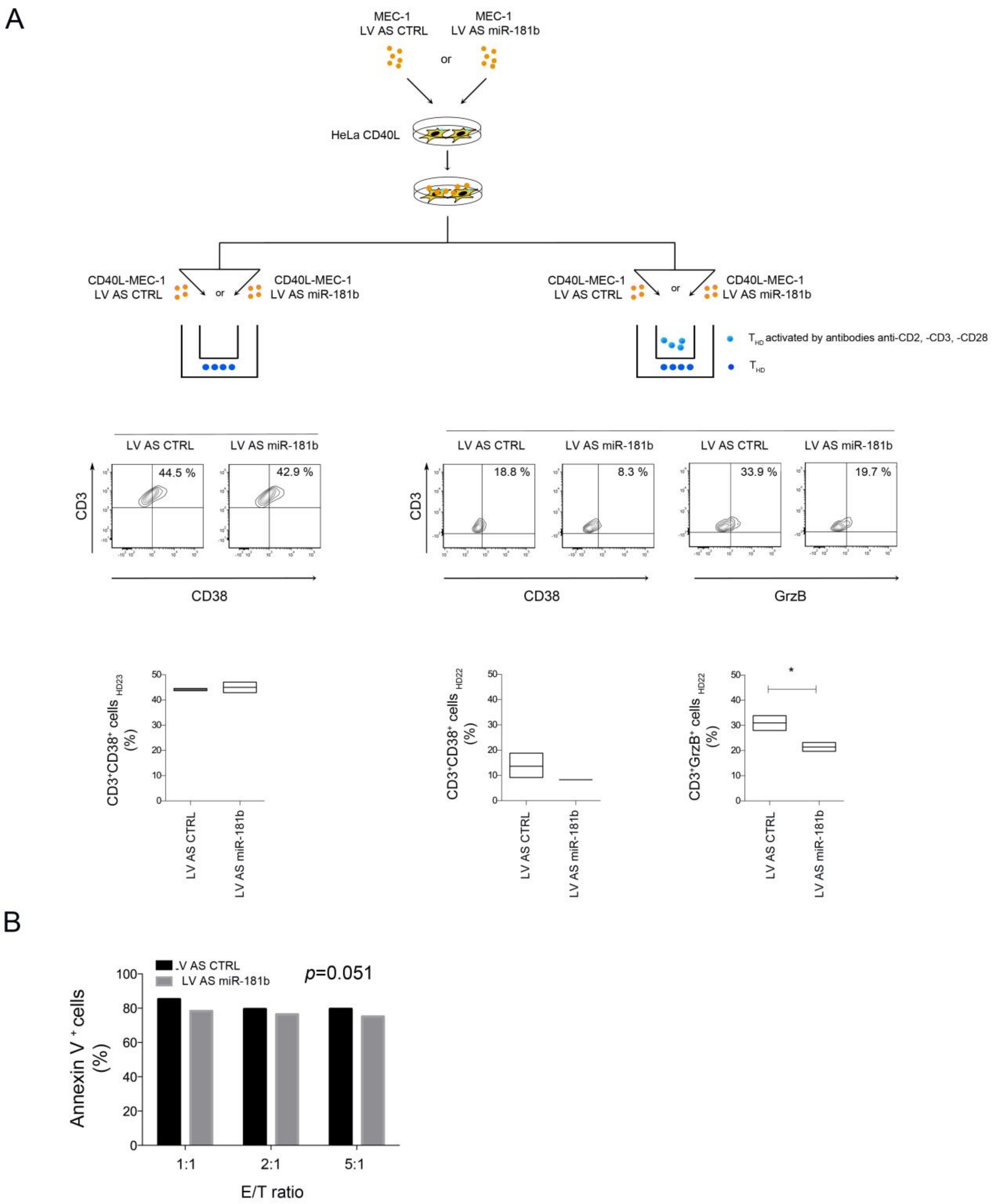

Figure S8. Depletion of *miR-181b* from B cells reduces T cell activation and maturation. (A) Percentage of CD3+/CD38+ and CD3+/GrzB+ from the lower transwell chamber. In the upper chamber, MEC-1 cells, infected with either LV AS miR181b or LV AS CTRL, were co-cultured with CD40L-HeLa. After 3 days, stimulated cells were grown with or without healthy activated T cells from healthy donor 22 (T_{HD22}). In the lower chamber were seeded T cells from healthy donors 22 (T_{HD22}) or 23 (T_{HD23}) as indicated. Greater maturation of T_{HD23} cells when directly mixed with MEC-1-LV AS CTRL than with LV AS miR181b was validated in a previous experiment (Fig.5B). Data are reported as floating bars (min to max) with the central line marking the mean of at least two

experimental replicates. *$p < 0.05$ by Student's t test. (B) Percentage of fresh MEC-1 cells VPD+/Ann+ after 2 h of co-culture. T cells from the healthy donor 22 (T_{HD22}) were mixed with CD40L-activated MEC-1 cells transduced with either or LV AS miR181b or LV AS CTRL (E/T ratio, 2:1); the CTLs generated were then mixed with third-part fresh MEC-1 cells (E/T ratios, 1:1, 2:1, 5:1). Each bar reports a single value. Significance was assessed using the paired *t* test.

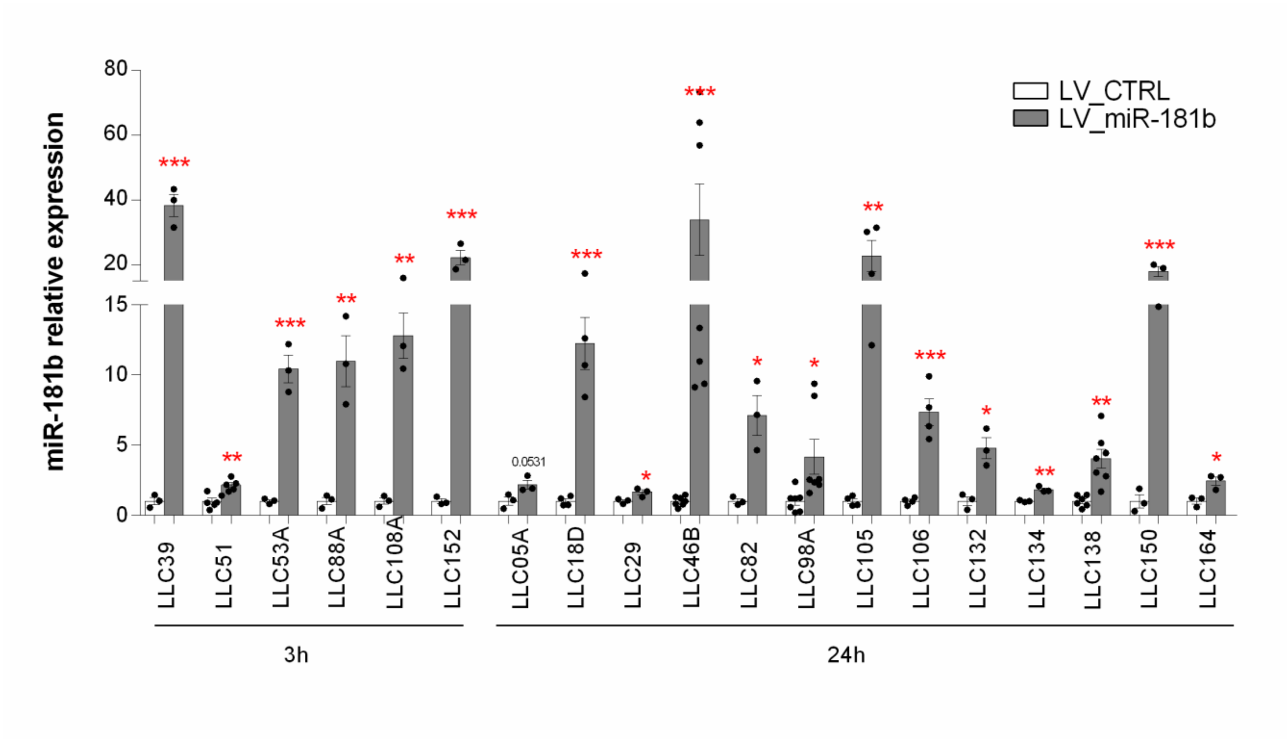

Figure S9. Evaluation of CLL cells infection. CLL cell infection was verified by RT-qPCR of *miR-181b* in purified CLL cells infected with LV miR-181b or LV CTRL; *miR-181b* expression was normalized to the endogenous references *RNU44* with the $2^{-\Delta ct}$ method. For each patient, the relative expression of *miR-181b* was normalized to the level of LV CTRL infected sample. Asterisks on each bar represents *p*-values calculated using Student's *t* test. Data are presented as mean ± SEM and technical replicates are shown for each sample as black dot. *$p < 0.05$, **$p < 0.01$ and ***$p < 0.001$.

.

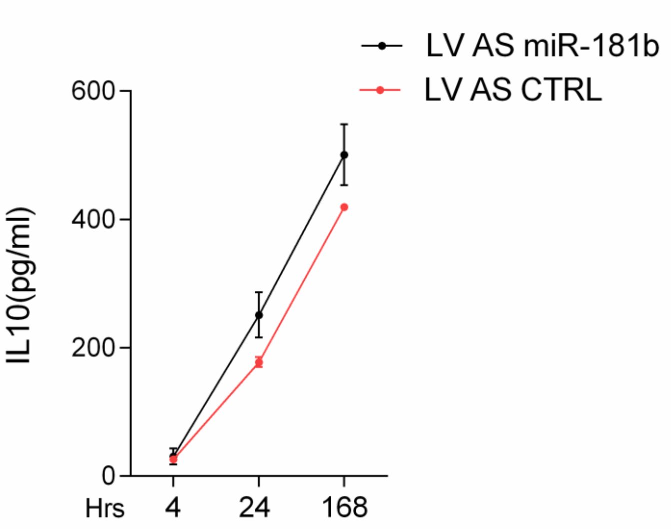

Figure S10. Depletion of *miR-181b* in CLL cell line increases IL10 secretion. ELISA determination of IL10 release in supernatant at 4, 24 and 168 hours from MEC-1 cells previously transduced with either LV AS miR-181b or LV AS CTRL and activated by HeLa cells expressing CD40L. Data are means ± SEM of experimental duplicates.

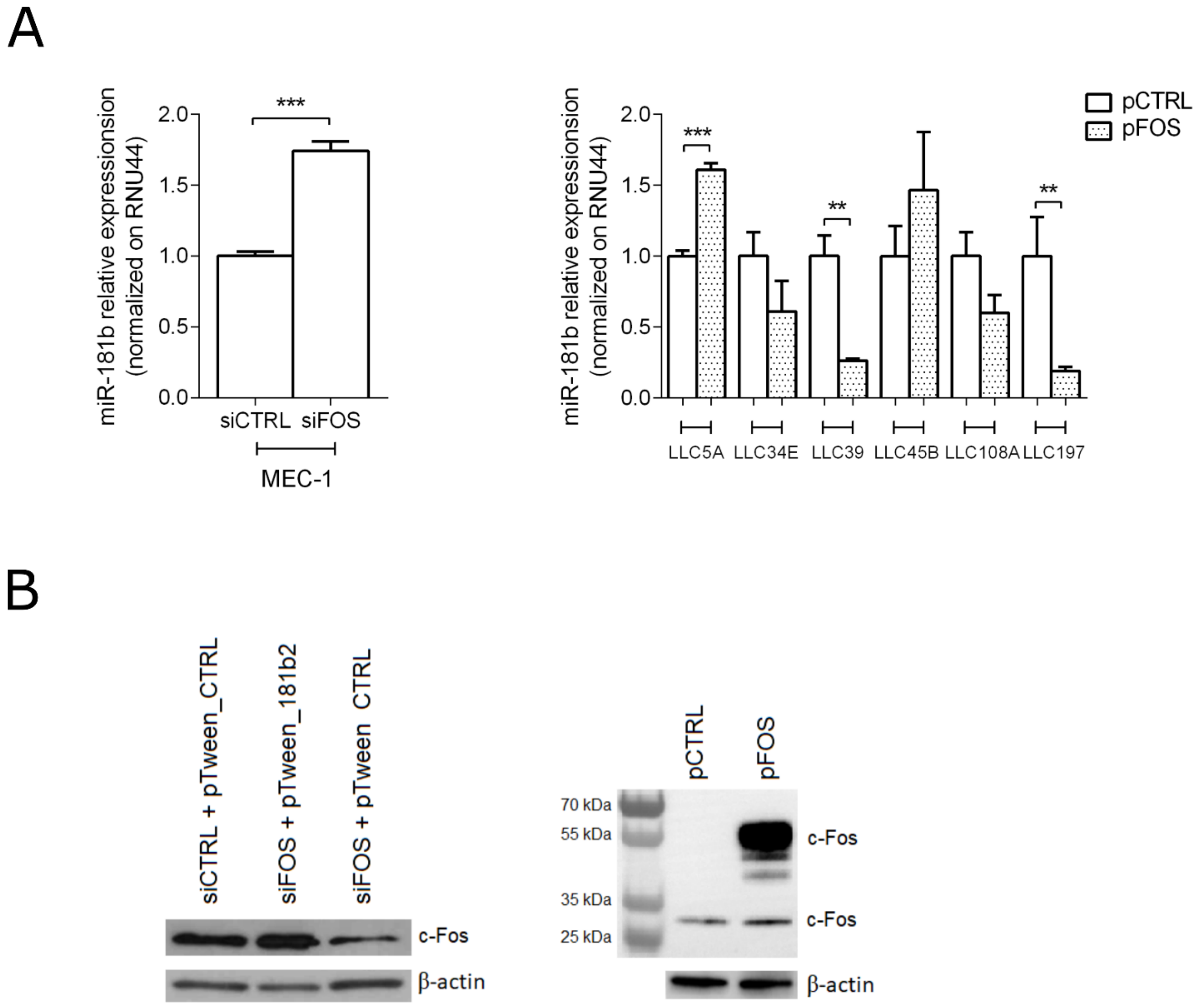

Figure S11. C-Fos protein regulates miR-181b expression. A) Relative expression of *miR-181b* by RT-qPCR in CLL cells after silencing (*left panel*) or overexpression (*right panel*) of c-Fos; *miR181b* expression was normalized to the endogenous reference *RNU44* with the $2^{-\Delta ct}$ method. Asterisks on each bar represents *p*-values calculated using Student's *t* test. B) Functional validation of siRNA-FOS (*left panel*) and pLX304-FOS-V5 (*right panel*, pFOS) in MEC-1 transfected cells; in our CLL cells, the molecoular weight of the endogenous c-Fos is between 25 and 35 kDa (PageRuler Plus, ThermoScientific), while the molecoular weight of the exogenous protein is around 55 kDa (PageRuler Plus, ThermoScientific). In the experiment reported in S11B left panel, the co-transfection of siFOS and pTween_181b2 increases c-Fos protein level, even though we expected a strong downregulation of c-Fos due to the synergic effect of *siFOS* and *miR-181b*. This could be due to a competition of the two RNAs for the binding of FOS-3'UTR, thus not allowing the right recruiting of the RISC complex.

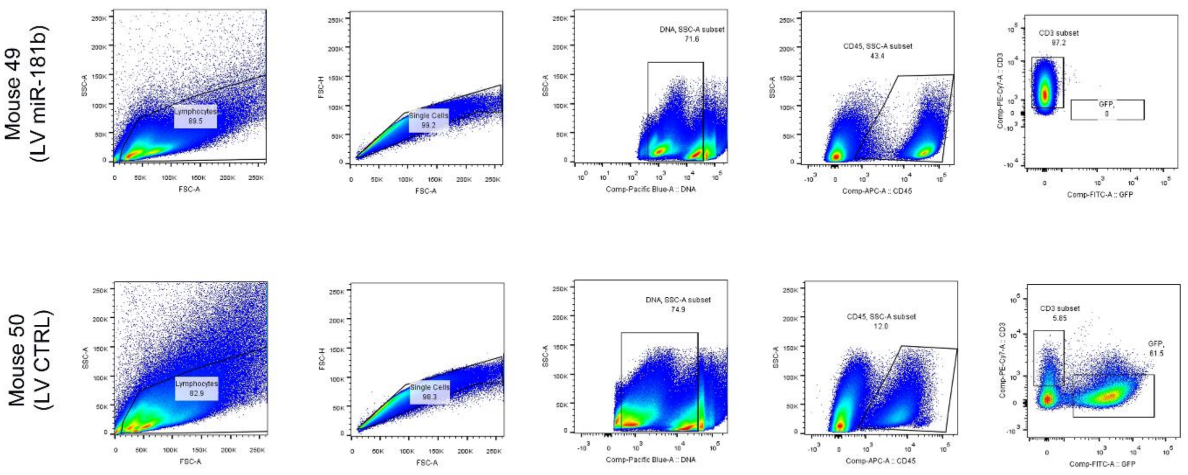

Figure S12. Flow cytometry gating strategy in *in vivo* experiments. Events showing the characteristic lymphocyte morphology were selected in a forward scatter (FSC-A) versus side scatter (SSC-A) plot (a). Next, single cells were identified (b) and dead cells were excluded based on the intensity staining of their nuclei (DNA positive, c). CD45 positive cell population was than gated (d) and analysed for CD3 surface expression and GFP (e). Representative plots of cells from bone marrow of mice inoculated with T cells and either MEC-1 carrying LV miR-181b (upper panel) or LV CTRL (lower panel).
